# Lnc-RP11-536 K7.3/SOX2/HIF-1α signaling axis regulates oxaliplatin resistance in patient-derived colorectal cancer organoids

**DOI:** 10.1186/s13046-021-02143-x

**Published:** 2021-11-05

**Authors:** Qingguo Li, Huizhen Sun, Dakui Luo, Lu Gan, Shaobo Mo, Weixing Dai, Lei Liang, Yufei Yang, Midie Xu, Jing Li, Peiyong Zheng, Xinxiang Li, Yan Li, Ziliang Wang

**Affiliations:** 1 Department of Colorectal Surgery, Fudan University Shanghai Cancer Center, 270 Dong’an Road, Shanghai, 200032 China; 2 Department of Oncology, Shanghai Medical College, Fudan University, Shanghai, 200032 China; 3 Clinical Medicine Transformation Center and Office of Academic Research, Shanghai Hospital of Traditional Chinese Medicine Affiliated to Shanghai University of Traditional Chinese Medicine, Shanghai, 200071 China; 4 Department of Obstetrics and Gynecology, Xinhua Hospital Affiliated to Shanghai Jiaotong University School of Medicine, Shanghai, 200092 China; 5 Department of Medical Oncology, Zhongshan Hospital, Fudan University, Shanghai, 200030 China; 6 Department of Pathology and Biobank, Fudan University Shanghai Cancer Center, Shanghai, 200032 China; 7 Department of CyberKnife Center, Huashan Hospital, Fudan University, Shanghai, 200040 China; 8 Institute of Digestive Diseases, Longhua Hospital, Shanghai University of Traditional Chinese Medicine, Shanghai, 200032 China; 9 Department of Biology, Southern University of Science and Technology, 1088 Xueyuan Blvd., Nanshan District, Shenzhen, 518055 China

**Keywords:** Lnc-RP11-536 K7.3, Organoid, Oxaliplatin, Colon cancer

## Abstract

**Background:**

Resistance to oxaliplatin is a major obstacle for the management of locally advanced and metastatic colon cancer (CC). Although long noncoding RNAs (lncRNAs) play key roles in CC, the relationships between lncRNAs and resistance to oxaliplatin have been poorly understood yet.

**Methods:**

Chemo-sensitive and chemo-resistant organoids were established from colon cancer tissues of the oxaliplatin-sensitive or -resistant patients. Analysis of the patient cohort indicated that lnc-RP11-536 K7.3 had a potential oncogenic role in CC. Further, a series of functional in vitro and in vivo experiments were conducted to assess the effects of lnc-RP11-536 K7.3 on CC proliferation, glycolysis, and angiogenesis. RNA pull-down assay, luciferase reporter and fluorescent in situ hybridization assays were used to confirm the interactions between lnc-RP11-536 K7.3, SOX2 and their downstream target HIF-1α.

**Results:**

In this study, we identified a novel lncRNA, lnc-RP11-536 K7.3, was associated with resistance to oxaliplatin and predicted a poor survival. Knockout of lnc-RP11-536 K7.3 inhibited the proliferation, glycolysis, and angiogenesis, whereas enhanced chemosensitivity in chemo-resistant organoids and CC cells both in vitro and in vivo. Furthermore, we found that lnc-RP11-536 K7.3 recruited SOX2 to transcriptionally activate USP7 mRNA expression. The accumulative USP7 resulted in deubiquitylation and stabilization of HIF-1α, thereby facilitating resistance to oxaliplatin.

**Conclusion:**

In conclusion, our findings indicated that lnc-RP11-536 K7.3 could promote proliferation, glycolysis, angiogenesis, and chemo-resistance in CC by SOX2/USP7/HIF-1α signaling axis. This revealed a new insight into how lncRNA could regulate chemosensitivity and provide a potential therapeutic target for reversing resistance to oxaliplatin in the management of CC.

**Supplementary Information:**

The online version contains supplementary material available at 10.1186/s13046-021-02143-x.

## Background

Colon cancer (CC) is the fifth most common malignancy and the fifth leading cause of cancer-related mortality among 36 types of cancer worldwide [1]. Oxaliplatin-based chemotherapy is recommended for locally advanced or metastatic CC [2, 3]. Acquisition of resistance to chemotherapy results in therapy failure and disease progression in a number of CC patients. Great efforts have been dedicated to reveal the mechanism of resistance acquisition of oxaliplatin and propose some interventional strategies [4, 5]. However, the cause of resistance acquisition of oxaliplatin remains elusive. A limited number of prognostic factors have been developed and validated as predictive biomarkers or therapeutic targets [6–9]. Thus, it is highly essential to elucidate the precise mechanisms of resistance to oxaliplatin, identify effective biomarkers for predicting oxaliplatin response, and develop targeted therapies for minimizing resistance to oxaliplatin in CC patients.

Long non-coding RNAs (lncRNAs) are RNA molecules, which are longer than 200 nucleotides without a protein coding potential. Emerging evidence has indicated that lncRNAs play significant roles in multiple physiological and pathological processes by distinctive mechanisms [10, 11]. Notably, aberrant expression of lncRNAs has been reported in diverse types of cancer and dysregulation of lncRNAs participates in tumorigenesis and chemotherapy resistance [12–14]. However, the influences of lncRNAs on resistance to oxaliplatin have been poorly understood. In the present study, we established oxaliplatin-resistant and -sensitive organoids derived from CC patients to identify an oxaliplatin-resistant-related lncRNA, namely lnc-RP11-536 K7.3. Functions and mechanisms of lnc-RP11-536 K7.3 were further dissected in CC. Our findings demonstrated that lnc-RP11-536 K7.3 could participate in resistance to oxaliplatin and could be a promising therapeutic target for chemosensitization in CC.

## Materials and methods

### Patients and tissue samples

Tissue microarrays were collected from patients with CC who were admitted to Fudan University Shanghai Cancer Center (FUSCC; Shanghai, China) from 2007 to 2009. Clinical data are summarized in Supplementary Table S1. Overall survival (OS) was measured as the length of time from initiation of surgery to death from any cause or the most recent follow-up. Progression-free survival (PFS) was calculated from the date of surgery to occurrence of progression or relapse. PFS less than 6 months was defined as resistant to the last chemotherapy; otherwise, it was defined as sensitive to the last chemotherapy.

### Collection and culture of organoids

Chemo-sensitive organoids in our study were collected from chemo-sensitive patients’ CC tissues in the first surgery. The chemo-resistant organoids were gained from CC patients, who underwent reoperation after failure of oxaliplatin-based chemotherapy. Then, the organoids were cultured as described below and drug-resistance testing was performed with oxaliplatin the treatment for 25 days.

After that, fresh tumor tissues were immediately cultured in advanced Dulbecco’s modified Eagle’s medium (DMEM)/F12 supplemented with 1% penicillin streptomycin. Tissues were diced into approximately 2–3-mm sections, and then, digested at 37 °C for 1 h by trypsin. The digested tissues were filtered through a 70-μm filter (catalog number: 352350; Falcon, San Diego, CA, USA). The cell suspension was then spun at 1000 rpm for 5 min to create a cell pellet. The pellet was washed with red blood for 2–3 times. Cells in solid tumor were then mixed with growth factor reduced Matrigel (catalog number: CB-40230C; Corning, New York, NY, USA), and cellular concentration was set to 10,000 ~ 20,000 cells/50 μL. Once the Matrigel was solidified, 500 μL of general (DMEM)/F12 culture medium was added. Patient-derived organoids (PDOs) were kept in a humidified atmosphere of 5% CO_2_ and 95% air at 37 °C, and medium was changed every 2 ~ 3 days. The splitting ratio was 1:3.

### Statistical analysis

Data were statistically analyzed by using GraphPad Prism software (GraphPad Software Inc., San Diego, CA, USA) and presented as mean ± SD. Comparisons between control and treatment groups were performed via the paired *t*-test or one-way analysis of variance (ANOVA), followed by Tukey multiple comparison tests. Clinicopathological data were analyzed using SPSS 24.0 software (IBM, Armonk, NY, USA). The Kaplan–Meier method and the log-rank test were employed to estimate OS. Variables with *P*-values < 0.05 in univariate analysis were included in subsequent multivariate analyses based on the Cox proportional-hazards model. A probability with value of less than 0.05 was considered statistically significant.

See online supplementary section for detailed explanation on methods.

## Results

### Lnc-RP11-536 K7.3 was highly expressed in oxaliplatin-resistant organoids of CC patients and predicted worse prognosis

In order to investigate mechanisms of resistance to oxaliplatin through a model with a superior genetic and phenotypic recapitulation, chemo-resistant and -sensitive organoids derived from CC patients were established. Representative images of hematoxylin-eosin (HE) staining of oxaliplatin-resistant and -sensitive human CC tissues are shown in Fig. S1A. As expected, oxaliplatin significantly inhibited the growth of oxaliplatin-sensitive organoid, whereas it slightly influenced oxaliplatin-resistant organoids (Fig. S1B-C). Representative images of HE staining of oxaliplatin-resistant and -sensitive organoids of human CC tissues are displayed in Fig. S1D. IHC revealed that the expressions of CK20 and β-catenin were higher in oxaliplatin-resistant organoids compared with oxaliplatin-sensitive organoids, indicating that oxaliplatin-resistant organoids behaved more aggressively (Fig. S1E). Consistently, the expressions of CK20 and β-catenin were higher in oxaliplatin-resistant human CC tissues compared with oxaliplatin-sensitive human CC tissues (Fig. S2A).

**Fig. 1:**
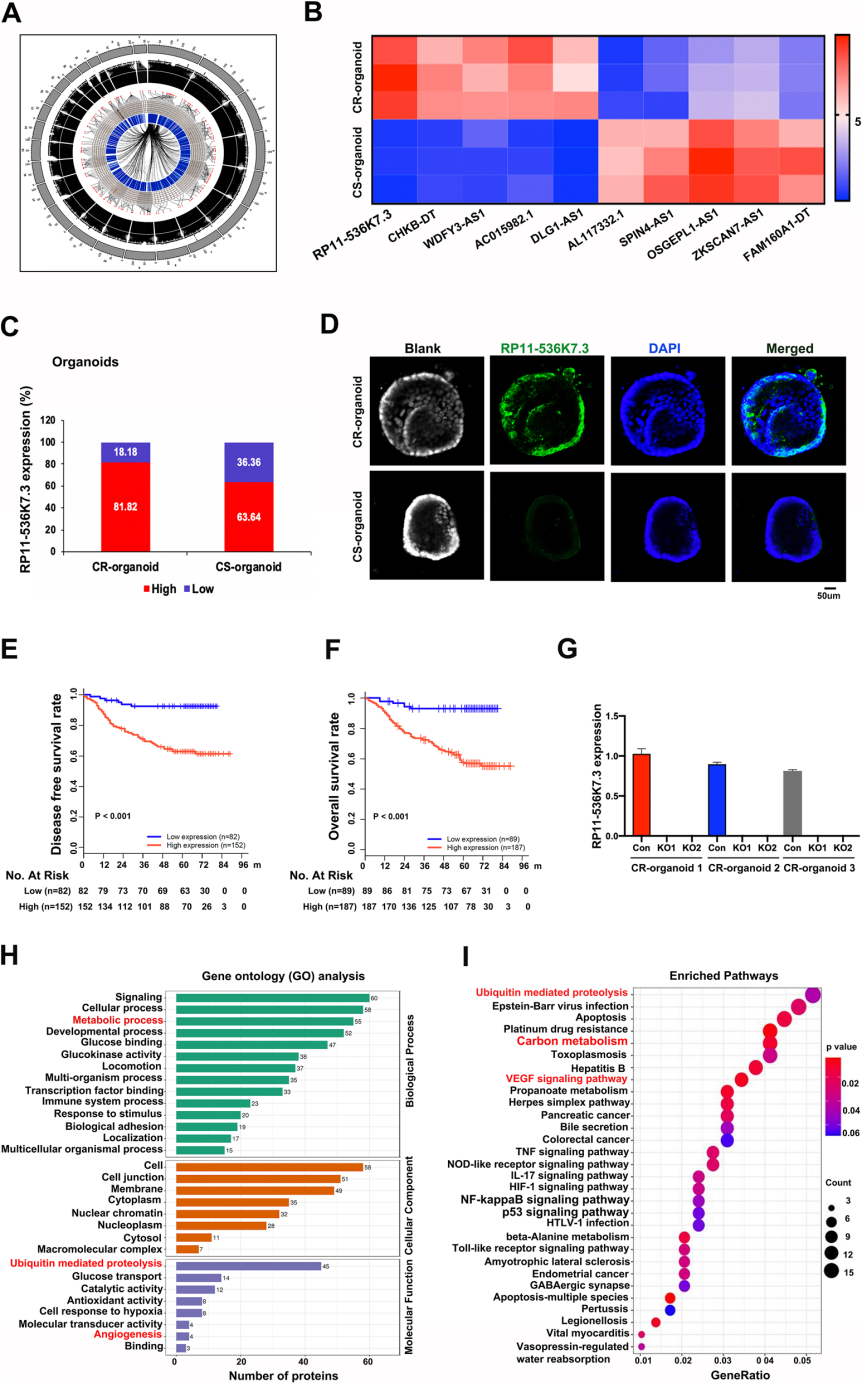
lnc-RP11-536 K7.3 was highly expressed in oxaliplatin-resistant organoids of CC patients. **A** Circos plot displaying the distribution and expression of lncRNAs on human chromosomes. The outermost layer was a chromosome map of the human genome. The inner circles from outside to inside were corresponded to distribution and expression of detected lncRNAs on the chromosomes, distribution and expression of significantly expressed lncRNAs, and predicted mRNAs sponged by significantly expressed lncRNAs, respectively. **B** Differentially expressed lncRNAs between 3 oxaliplatin-resistant and 3 -sensitive organoids. **C** Differential expression of lnc-RP11-536 K7.3 in 22 oxaliplatin-resistant and 22 -sensitive organoids of CC patients detected by qPCR. The experiment was repeated 3 times. **D** Fluorescence in situ hybridization (FISH) assay of lnc-RP11-536 K7.3 in 22 oxaliplatin-resistant and 22 -sensitive organoids of CC patients. **E** Kaplan-Meier plot of disease free survival (DFS) according to lnc-RP11-536 K7.3 expression. Data were obtained from Shanghai Cancer Center. **F** Kaplan-Meier plot of overall survival (OS) according to lnc-RP11-536 K7.3 expression. Data were obtained from Shanghai Cancer Center. **G** qRT-PCR assay was used to detect the efficacy of lnc-RP11-536 K7.3 knockout in oxaliplatin-resistant organoids. The experiment was repeated 3 times. **H** Gene ontology of mass spectrum analysis in oxaliplatin-resistant and -sensitive colon cancer organoids. **I** Pathway examination of mass spectrum analysis in oxaliplatin-resistant and -sensitive colon cancer organoids

To gain a deep insight into the molecular mechanism and dysregulated pathways of oxaliplatin resistance, genome-wide analysis of lncRNA expression was carried out in 3 oxaliplatin-resistant and 3 -sensitive organoids of CC patients. Circos plot was employed to display distribution and expression of lncRNAs on human chromosomes (Fig. 1A). It was found that lnc-RP11-536 K7.3 was among the most significantly differentially expressed lncRNAs in the two groups (Fig. 1B). Then, we confirmed that lnc-RP11-536 K7.3 was up-regulated in oxaliplatin-resistant organoids compared with oxaliplatin-sensitive organoids by qRT-PCR analysis (Fig. 1C). Fluorescence in situ hybridization (FISH) assay further approved that lnc-RP11-536 K7.3 was mainly expressed in oxaliplatin-resistant organoids of CC patients (Fig. 1D). We also evaluated the expression of lnc-RP11-536 K7.3 in Tissue microarray (TMA) consisting of 276 patients. High expression of lnc-RP11-536 K7.3 was detected in 67.75% of the patients (Table S1). Kaplan-Meier survival analysis revealed that significantly shorter overall survival (OS) and disease-free survival (DFS) were associated with high expression of lnc-RP11-536 K7.3 in CC patients (Fig. 1E-F). Subsequently, we also found that the expression of lnc-RP11-536 K7.3 was up-regulated in different cancer tissues compared with adjacent non-tumor tissues using date acquired from The Cancer Genome Atlas (TCGA) and high expression of lnc-RP11-536 K7.3 predicted a poor prognosis (Fig. S2B-C). We selected three oxaliplatin-resistant organoids to knockout expression of lnc-RP11-536 K7.3 for further analysis (Fig. 1G). Using Gene Ontology (GO) analysis, we found that the genes associated with resistance to oxaliplatin resulted by the knockout of lnc-RP11-536 K7.3 were enriched in metabolic process, ubiquitin-mediated proteolysis, and angiogenesis (Fig. 1H). Kyoto Encyclopedia of Genes and Genomes (KEGG) pathway analysis also revealed that ubiquitin-mediated proteolysis, carbon metabolism, and vascular endothelial growth factor (VEGF) signaling pathway were involved in resistance to oxaliplatin (Fig. 1I).

### Silencing of lnc-RP11-536 K7.3 repressed resistance to oxaliplatin in CC samples

**Fig. 2:**
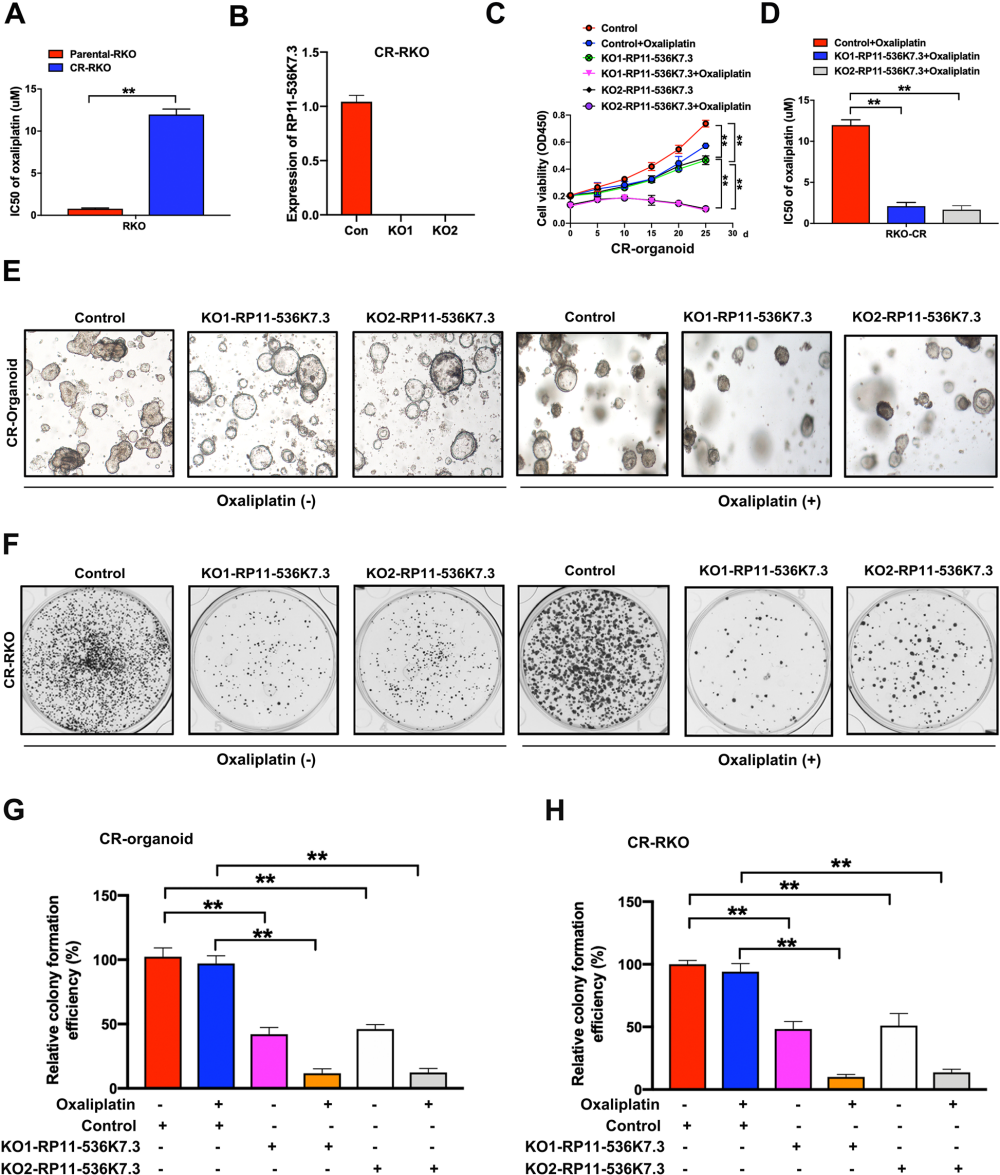
Knockout of lnc-RP11-536 K7.3 increased chemosensitivity in chemo-resistant organoids and cells in CC patients. **A** Chemo-resistant RKO (CR-RKO) cell line was generated by gradual increase of oxaliplatin concentration. CCK8 assay showed that IC50 value of CR-RKO cells was much higher than that of the parental RKO cells (^**^P < 0.01). **B** qRT-PCR assay was used to detect the efficacy of lnc-RP11-536 K7.3 knockout in CR-RKO cell lines. **C** Cell viability assay of organoids treated with or without 1 uM oxaliplatin in different time intervals. **D** IC50 values of oxaliplatin. Lnc-RP11-536 K7.3-knockout and control cells were treated with different concentrations of oxaliplatin for 48 h (^**^P < 0.01). **E-H** Colony formation efficiency of chemo-resistant CC organoids and cells treated with or without 2 uM oxaliplatin for 7 days. Represent pictures in chemo-resistant CC organoiI (**E**) and chemo-resistant RKO cells (**F**). Statistical analysis of relative colony formation in chemo-resistant CC organoids (**G**) and chemo-resistant RKO cells (**H**) (^**^P < 0.01). Above experiments were repeated 3 times

Using patient-derived organoids and drug-resistant tumor cell line models, we attempted to determine the influence of lnc-RP11-536 K7.3 on resistance to oxaliplatin in a robust manner. Oxaliplatin-resistant RKO (CR-RKO: Chemo-resistant RKO) CC cell line was established by treating cells with increasing concentrations of oxaliplatin. Compared with parental cells, CR-RKO cells responded poorly to oxaliplatin, as evidenced by an increased half maximal inhibitory concentration (IC50) value (Fig. 2A). We then knocked out the expression of lnc-RP11-536 K7.3 in CR-RKO cells to examine its role in cell viability (Fig. 2B). Through CCK-8 assay we discovered that depletion of lnc-RP11-536 K7.3 significantly decreased viability of oxaliplatin-resistant CC organoids and increased sensitivity to oxaliplatin treatment in models of organoids and cells (Fig. 2C-D). Subsequent colony formation efficiency of chemo-resistant CC organoids and CR-RKO cells was also obviously reduced after silencing of lnc-RP11-536 K7.3 (Fig. 2E-H). Intriguingly, the suppressive effects of lnc-RP11-536 K7.3 depletion on colony formation were markedly more evident under oxaliplatin treatment, suggesting that oxaliplatin resistant CC cells were associated with lnc-PR11-536 K7.3 expression. Above results indicated that lnc-RP11-536 K7.3 induced resistance to oxaliplatin in CC.

### Lnc-RP11-536 K7.3 increased glycolysis and angiogenesis in CC

**Fig. 3:**
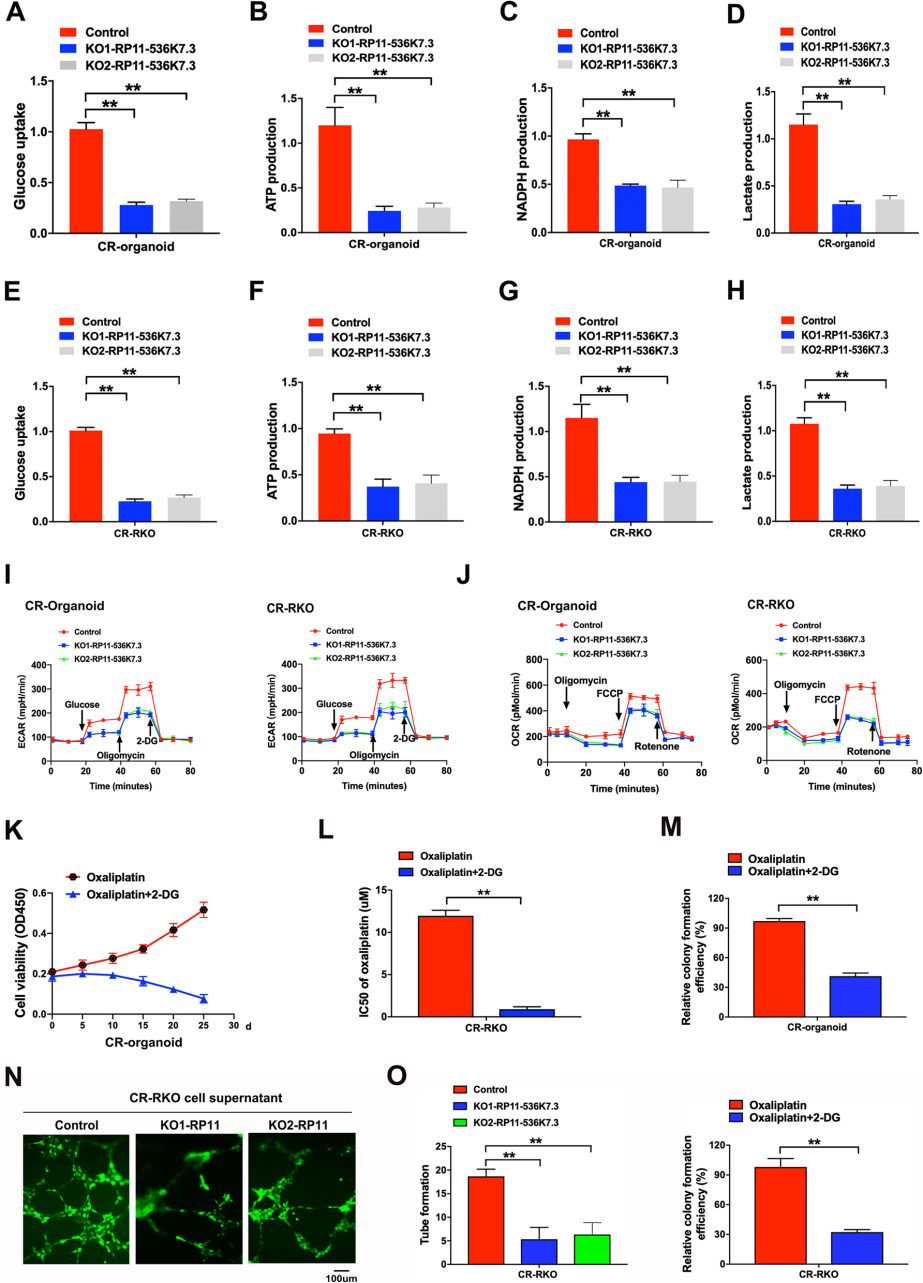
Knockout of lnc-RP11-536 K7.3 attenuated glycolysis and angiogenesis. **A-H** Determination of glucose uptake (**A** and **E**), ATP (**B** and **F**), NADPH (**C** and **G**), and lactate production (**D** and **H**) in CC organoids and cells as described in Methods. Data were presented as mean ± SD of triplicate measurements repeated three times with similar results. Statistical significance was assessed via the Student’s t-test(^**^P < 0.01). **I-J** Measurement of ECAR (**I**) and OCR (**J**) in CC organoids and cells as described in Methods. **K** Cell viability assay of organoids treated with 1 uM oxaliplatin alone or in combination with 2.5 mM 2-DG in different time intervals. **L** IC50 values of cisplatin. CC cells were treated with different concentrations of oxaliplatin with or without 2-DG (5 mM for 48 h) (^**^P < 0.01). **M** Colony formation efficiency of chemo-resistant CC organoids and cells treated with 2 uM oxaliplatin alone or in combination with 2.5 mM 2-DG for 7 days (^**^P < 0.01). **N-O** Effects of knockout of lnc-RP11-536 K7.3 on HUVECs. HUVECs were treated with supernatant obtained from CR-RKO/KO1-RP11-536 K7.3, CR-RKO/KO2-RP11-536 K7.3 or the corresponding control cells. Represent pictures of different groups (**N**). Statistical analysis of tube formation and relative colony formation effciency (^**^P < 0.01) (**O**). Above experiments were repeated 3 times

In the current study, proteomic analysis of organoids revealed that glycolysis and angiogenesis were closely correlated with chemo-resistance [15]. Therefore, we determined whether lnc-RP11-536 K7.3 could regulate glycolysis in chemo-resistant CC organoids and cells. Lnc-RP11-536 K7.3 depletion decreased glycolysis and mitochondrial oxidative phosphorylationin chemo-resistant CC organoids and cells, as evidenced by a reduced glucose uptake, ATP, nicotinamide adenine dinucleotide phosphate (NADPH), ECAR, and OCR (Fig. 3A-J). We further examined whether the inhibition of glycolysis could mimic the role of lnc-RP11-536 K7.3 depletion in oxaliplatin resistance. As expected, 2-deoxyglucose, a glycolytic inhibitor, diminished resistance to oxaliplatin and decreased IC50 of oxaliplatin (Fig. 3K-M). In addition to glycolysis, we also investigated the role of lnc-RP11-536 K7.3 knockout in angiogenesis. Human umbilical vein endothelial cells (HUVEC) cultured in the conditional medium of lnc-RP11-536 K7.3-depleted CR-RKO cells formed significantly less tubes (Fig. 3N-O). Moreover, we constructed lnc-RP11-536 K7.3 overexpressed CC organoids and cells in the lnc-RP11-536 K7.3 depleted organoids and cells, and found that lnc-RP11-536 K7.3 overexpression effectively reversed the decrease of glycolysis level and tube formation induced by lnc-RP11-536 K7.3 knockout (Fig. S3A-G).

### Lnc-RP11-536 K7.3 recruited SOX2 to transcriptionally activate deubiquitinase USP7 and subsequently enhanced HIF-1α stability

**Fig. 4:**
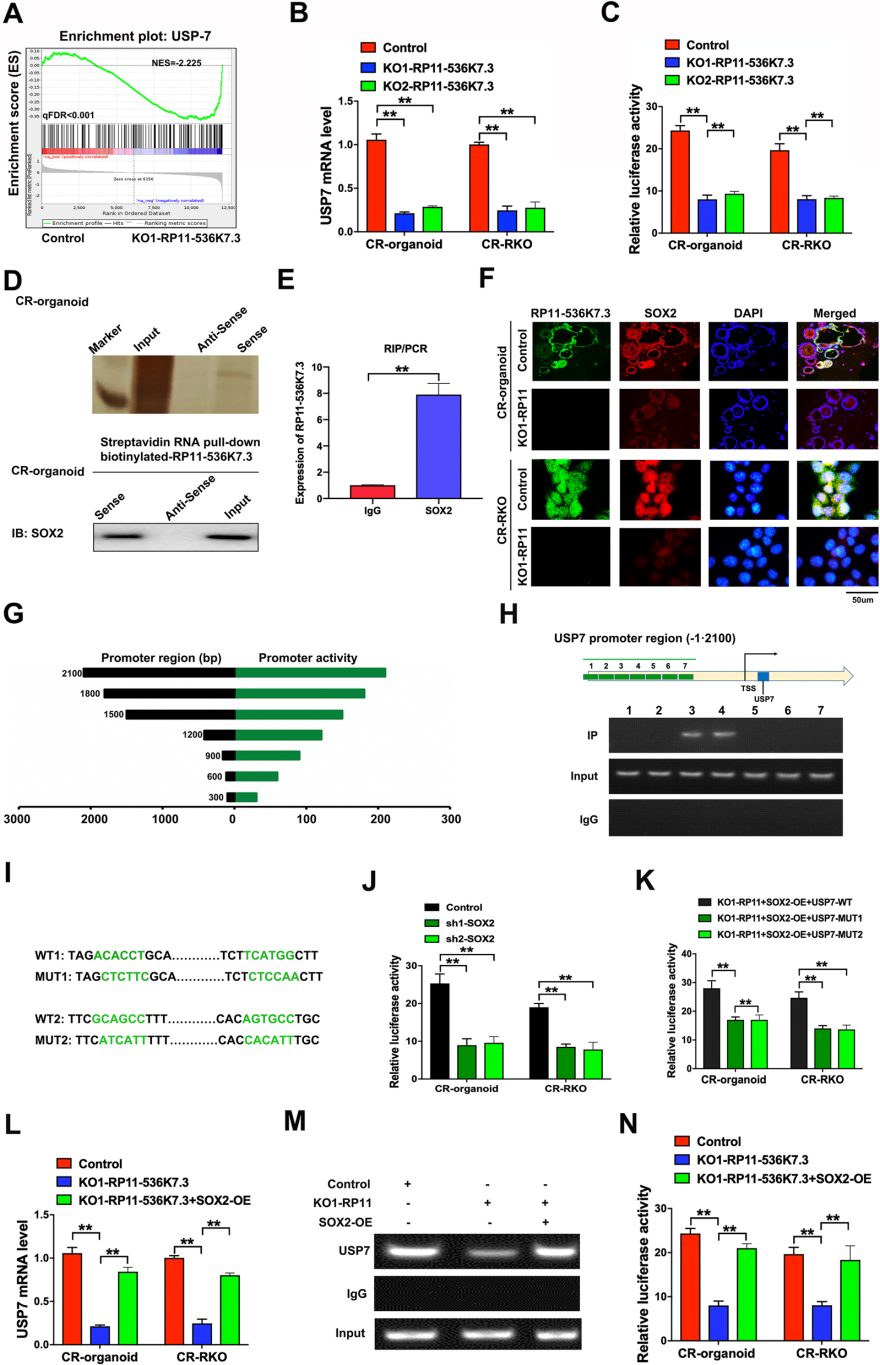
lnc-RP11-536 K7.3 recruit SOX2 to regulate the promoter activity of USP7. **A** GSEA was performed in lnc-RP11-536 K7.3-KO organoids and control group. The gene signature was defined by genes with significant expression changes. **B** qRT-PCR assay of USP7 mRNA in lnc-RP11-536 K7.3-KO organoids and cells and control groups (^**^P < 0.01). The experiment was repeated 3 times. **C** Luciferase reporter assay indicated that USP7 activity was affected by knockout of lnc-RP11-536 K7.3 (^**^P < 0.01). The experiment was repeated 3 times. **D** RNA pull-down assay followed by silver staining and western blot was performed on chemo-resistant CC organoids. **E** qRT-PCR was carried out after completion of RNA RIP assay. The experiment was repeated 3 times. **F** FISH and immunofluorescence assay detected the expressions of lnc-RP11-536 K7.3 and SOX2 in lnc-RP11-536 K7.3 knocked out organoids and CR-RKO cell lines and controls. The experiment was repeated 3 times. **G-H** The map of SOX2 binding sits in the promoter region of USP7 and the results of ChIP analysis showed that SOX2 can bind to the USP7 promoter region. **I-K** Luciferase reporter assay of SOX2 mutant sites in the promoter region of USP7. (^**^P < 0.01). The experiment was repeated 3 times. **L** qRT-PCR assay of USP7 mRNA in knockout of lnc-RP11-536 K7.3 and overexpression of SOX2 organoids and cells and control groups (^**^P < 0.01). The experiment was repeated 3 times. **M** CHIP results showed that SOX2 could bind to USP7 and mutation of SOX2 binding motifs abrogated its transcriptional regulation on USP7. **N** Luciferase reporter assay indicated that USP7 activity was affected by knockout of lnc-RP11-536 K7.3 and overexpression of SOX2 (^**^P < 0.01). The experiment was repeated 3 times

In order to interrogate the activity and mechanism of lnc-RP11-536 K7.3, we performed high-throughput RNA sequencing on lnc-RP11-536 K7.3 knocked out organoids. It was noted that lncRNAs could influence the expressions of neighboring genes [16]. As no neighboring genes were detected with differential expression, we postulated that lnc-RP11-536 K7.3 might execute functions in the transcriptional level. To determine the downstream target of lnc-RP11-536 K7.3, we compared the expression profile between lnc-RP11-536 K7.3 knocked out organoids and control group, which revealed that USP7 was positively correlated with lnc-RP11-536 K7.3 expression at mRNA level (Fig. 4A). Besides, the results of qRT-PCR assay found that lnc-RP11-536 K7.3 depletion resulted in a notable downregulation of USP7 (Fig. 4B). Luciferase reporter assay also indicated that USP7 promoter activity was affected by the knockout of lnc-RP11-536 K7.3 (Fig. 4C). The above results indicated that lnc-RP11-536 K7.3 might increase the expression of USP7 by enhancing the activity of USP7 promoter.

To explore the underlying regulatory mechanism of lnc-RP11-536 K7.3 on the promoter activity of USP7, we conducted RNA pull-down assay and mass spectrometry analysis to search for the potential mediators binding to lnc-RP11-536 K7.3 using chemo-resistant CC organoids. The results indicated that lnc-RP11-536 K7.3 specifically pulled down SOX2 protein (Fig. 4D). Furthermore, RIP assay was conducted to validate this finding and found a remarkable enrichment of lnc-RP11-536 K7.3 using an anti-SOX2 antibody compared with using IgG (Fig. 4E). These results indicated that lnc-RP11-536 K7.3 interacted with the SOX2 protein. FISH and immunofluorescence assay showed that the expression level of SOX2 was positively associated with that of lnc-RP11-536 K7.3 in chemo-resistant CC organoids and CR-RKO cell lines (Fig. 4F). Moreover, the enhanced expression of SOX2 could significantly increase cell viability inhibition induced by lnc-RP11-536 K7.3 depletion in chemo-resistant CC organoids, as well as IC50 of chemo-resistant cells upon oxaliplatin treatment (Fig. S4A-B). Similarly, overexpression of SOX2 partially counteracted the inhibitory effect of lnc-RP11-536 K7.3 depletion on colony formation efficiency in both chemo-resistant CC organoids and CR-RKO cell lines treated with or without oxaliplatin (Fig. S4C). Additionally, overexpression of SOX2 partially neutralized the inhibitory effect of lnc-RP11-536 K7.3 depletion on glycolysis in chemo-resistant CC organoids and CR-RKO cell lines (Fig. S4D-F).

By the analysis of RNA-Sequencing assay, we found that deubiquitinating enzyme USP7 was a potential target gene of SOX2. To confirm the exact region within the USP7 promoter that SOX2 binds, we performed ChIP assays and identified two SOX2 binding sites existed at approximately 900–1500 bp upstream of the open reading frame of USP7. (Fig. 4G-I) Mutation of each SOX2 binding motif abrogated its transcriptional regulation on USP7 (Fig. 4J-K). qRT-PCR assay revealed that lnc-RP11-536 K7.3 depletion resulted in a notable downregulation of USP7 while overexpression of SOX2 reversed the inhibitory effect of lnc-RP11-536 K7.3 depletion on USP7 (Fig. 4L). Coexistence of lnc-RP11-536 K7.3 strengthened the binding of SOX2 to USP7, indicating that interaction between lnc-RP11-536 K7.3 and SOX2 has a synergetic effect on USP7 activation (Fig. 4M). Luciferase reporter assay also indicated that the overexpression of SOX2 reversed the effect of knockout of lnc-RP11-536 K7.3 on USP7 activity (Fig. 4N). These results suggested that lnc-RP11-536 K7.3 can recruit SOX2 to regulate the promoter activity of USP7.

**Fig. 5:**
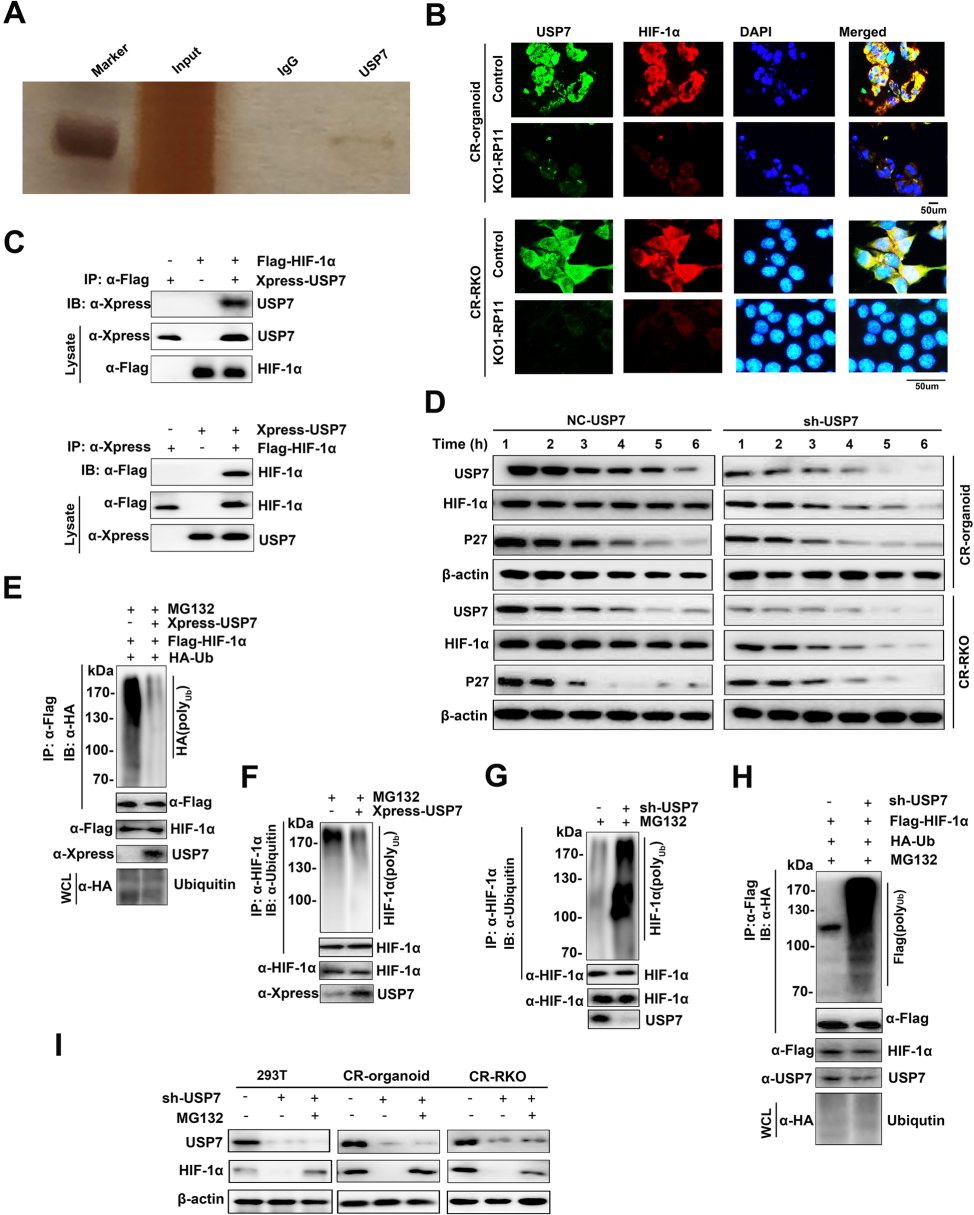
lnc-RP11-536 K7.3 combined with SOX2 to modulate HIF-1α stability by USP7. **A** Immunoprecipitation and mass spectrometry analysis identified HIF-1α was a potential USP7-interacting protein in HEK293T cells. **B** FISH and immunofluorescence assay detected the expressions of USP7 and HIF-1α in lnc-RP11-536 K7.3 knocked out organoids and CR-RKO cell lines and controls. **C** HIF-1α interacts with USP7. Flag-HIF-1α and Xpress-USP7 plasmids were co-transfected into HEK293T, and the interaction between HIF-1α and USP7 was determined by immunoprecipitation with α-Flag beads (top), or α-Xpress beads (bottom) followed by immunoblotting with α-Xpress or α-Flag antibody. One percent of the whole cell lysates was loaded as input control. **D** Stability of HIF-1α was reduced by silencing of USP7. Chemo-resistant CC organoids and cells transfected with shUSP7 were treated with cycloheximide, and collected at the indicated times for western blot. **E** USP7 deubiquitinates HIF-1α in cells. Xpress-USP7, Flag-HIF-1α, and HA-Ub plasmids were co-transfected into HEK293T cells. The ubiquitination of precipitated HIF-1α was analyzed by immunoblotting with anti-HA antibody. **F** USP7 deubiquitinates endogenous HIF-1α. Xpress-USP7 plasmids were transfected into HEK293T cells, and ubiquitination of precipitated endogenous HIF-1α was analyzed by immunoblotting with anti-ubiquitin antibody. **G** Knockdown of USP7 promotes ubiquitination of endogenous HIF-1α. Endogenous HIF-1α was immunoprecipitated from HEK293TshCtr or HEK293T-shUSP7 cells pretreated with MG132 (20 μmol/L). The ubiquitination of HIF-1α was analyzed by immunoblotting with anti-ubiquitin antibody. **H** Knockdown of USP7 promotes ubiquitination of HIF-1α. Flag-HIF-1α and HA-Ub plasmids were co-transfected into HEK293T-shCtr or HEK293T-shUSP47 cells, and cells were treated with MG132 (20 μmol/L). The ubiquitination of precipitated HIF-1α was analyzed by immunoblotting with anti-HA antibody. **I** Knockdown of USP7 promotes degradation of HIF-1α. HEK293T-shUSP7, CR-organoid-shUSP47, CR-RKO-shUSP47 and their controls were treated with or without MG132 (20 μmol/L). The expression levels of USP7, HIF-1α, and actin were detected. Above experiments were repeated 3 times

To further explore putative substrates of USP7, we generated HEK293T cells overexpressing HA-USP7 to perform immunoprecipitation (IP) and mass spectrometry analysis and found that HIF-1α was a potential USP7-interacting protein (Fig. 5A). As expected, the expressions of USP7 and HIF-1α exhibited a consistent trend, with reduced expression levels in lnc-RP11-536 K7.3 knocked out organoids and CR-RKO cell lines (Fig. 5B). Co-IP assay further revealed that USP7 primarily interacted with HIF-1α (Fig. 5C). In support of a role of USP7 in regulating HIF-1α stability, the half-life of HIF-1α was markedly shortened upon downregulation of USP7 using shRNA (Fig. 5D). Furthermore, we found that co-transfection USP7 specifically decreased ubiquitination of HIF-1α, while co-transfection of shRNAs targeting USP7 promoted ubiquitination of HIF-1α (Fig. 5E, H). As expected, ectopically expressed USP7 decreased ubiquitination of endogenous HIF-1α, while depletion of USP7 promoted ubiquitination of endogenous HIF-1α (Fig. 5F-G). These studies further suggested that HIF-1α is a possible physiologic substrate of USP7. Depletion of endogenous USP7 resulted in a significant decrease in expression level of endogenous HIF-1α in 293 T cell lines, CR-organoids, and CR-RKO cell lines, which could be further blocked by MG132 (Fig. 5I). Collectively, the above-mentioned results supported the notion that HIF-1α is a putative substrate of USP7.

### Knockout of lnc-RP11-536 K7.3 inhibited progression of CC and sensitized response to oxaliplatin in vivo

**Fig. 6:**
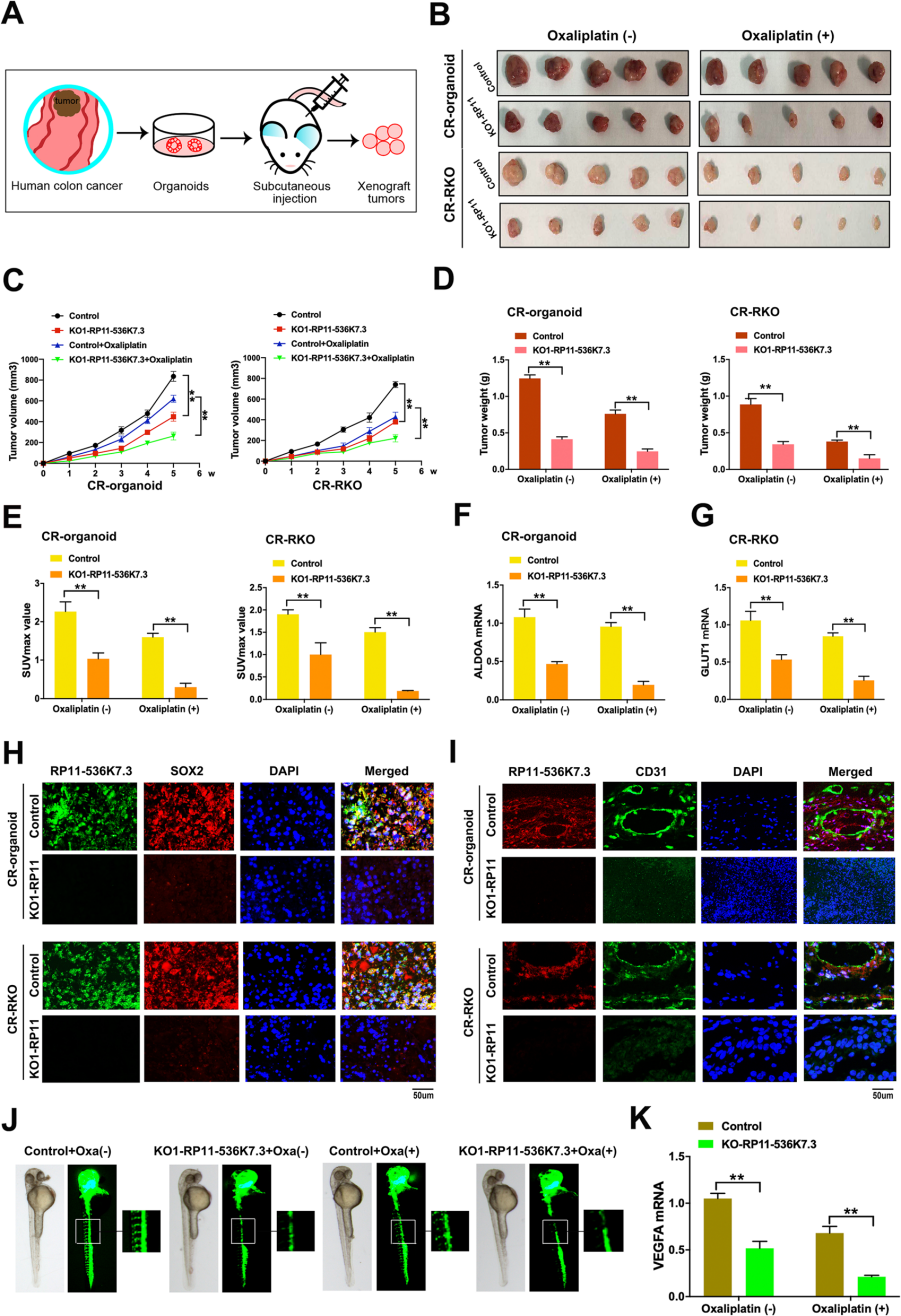
Knockout of lnc-RP11-536 K7.3 inhibits progression of CC cells and sensitizes response to oxaliplatin in vivo. **A** Schematic illustration of subcutaneous injection of the organoids and cells. **B** Representative images of nude mice bearing tumors generated by knockout of lnc-RP11-536 K7.3 and chemo-resistant CC organoids with or without oxaliplatin treatment. **C** Xenograft tumor growth in mice (^**^P < 0.01). **D** Average tumor weight of nude mice (^**^P < 0.01). **E** Average SUVmax value of PET-CT in nude mice bearing tumors. **F-G** qRT-PCR assay was used to detect the expressions of ALDOA and GLUT1. The experiment was repeated 3 times. **H-I** FISH and immunofluorescence assay were performed on the xenograft tumors of RP11-536 K7.3-KO and control groups with oxaliplatin treatment. **J** A zebrafish model treated with or without oxaliplatin. **K** qRT-PCR assay was employed to detect the expression of VEGFA in a zebrafish model (^**^P < 0.01). The experiment was repeated 3 times

To further evaluate the effects of lnc-RP11-536 K7.3 on tumorigenesis and chemo-resistance in vivo, lnc-RP11-536 K7.3 knocked out chemo-resistant CC organoids, CR-RKO cell lines, and control groups were then injected subcutaneously into nude mice (Fig. 6A). Mice bearing tumors were randomly grouped with administration of PBS or oxaliplatin. The volume and weight of tumor in the lnc-RP11-536 K7.3 knocked out group were significantly lower than those in the control group regardless of receiving PBS or oxaliplatin treatment (Fig. 6B-D). Of note, differences between lnc-RP11-536 K7.3 knocked out and control groups were noticeably more significant after administration of oxaliplatin compared with those that received PBS. Similarly, positron emission tomography/computed tomography (PET/CT) revealed that tumors derived from the lnc-RP11-536 K7.3 knocked out group exhibited lower mean values of SUVmax than the control group, indicating a reduced glucose uptake (Fig. 6E). The reductions were even more significant under oxaliplatin treatment. The expressions of ALDOA and GLUT1, two essential genes in the glycolysis pathway, were accordantly downregulated after knockout of lnc-RP11-536 K7.3 and the degree of downregulation was more obvious in group of treatment with oxaliplatin (Fig. 6F-G). Subsequently, we performed immunofluorescence in tissues derived from xenograft mice above, and discovered that lnc-RP11-536 K7.3 knockout group exhibited lower expressions of SOX2, USP7 and vascular endothelial marker CD31 compared with the control group with oxaliplatin treatment (Fig. 6H-I and Fig. S5A-B). Furthermore, a zebrafish model, a robust model in the study of angiogenesis, was exploited to verify the causal relationship between lnc-RP11-536 K7.3 and vascularization. In addition, knockout of lnc-RP11-536 K7.3 resulted in defective blood vascular patterning, which was especially significant under oxaliplatin treatment (Fig. 6J-K). The above-mentioned results demonstrated that knockout of lnc-RP11-536 K7.3 efficiently sensitized xenografts to oxaliplatin treatment in CC, accompanied by low level of glycolysis and vascular formation.

**Fig. 7:**
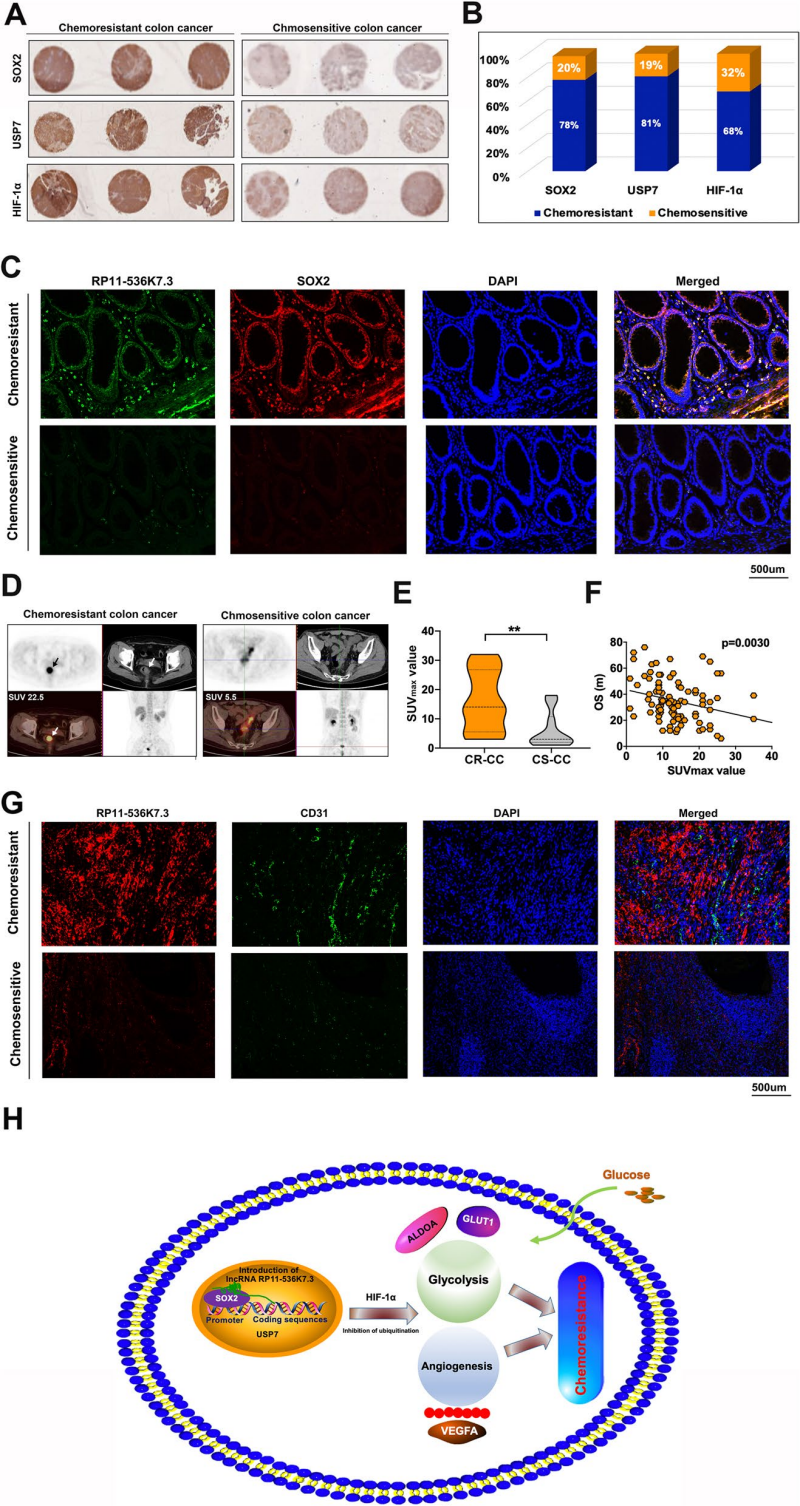
The expressions of SOX2, USP7, and HIF-1α in chemo-resistant and -sensitive CC tissues and the role of lnc-RP11-536 K7.3 in glucose uptake and angiogenesis. **A** Representative images of immunohistochemistry of SOX2, USP7, and HIF-1α in CC tissues. **B** The high expressions of SOX2, USP7, and HIF-1α in 168 oxaliplatin-resistant and 182 oxaliplatin-sensitive human colon cancer tissues. **C** FISH and immunofluorescence assay were conducted on human chemo-resistant and -sensitive CC tissues. Thirty pairs of human CC tissues were used. **D** Representative images of PET/CT in human chemo-resistant and -sensitive CC tissues. **E-F** The relationship between SUVmax value of PET/CT image and lnc-RP11-536 K7.3 expression. 100 colon cancer patients were tested in this experiment. **G** FISH and immunofluorescence assay were carried out on human chemo-resistant and -sensitive CC tissues. **H** Schematic illustration of the role of the lnc-RP11-536 K7.3/SOX2/USP7/HIF-1α signaling axis in regulation of glycolysis, angiogenesis, and sensitivity to oxaliplatin

To establish a clinical relevance in human CC, we analyzed the levels of lnc-RP11-536 K7.3, SOX2, USP7, and HIF-1α in human chemo-resistant and chemo-sensitive CC samples. High expressions of SOX2, USP7, and HIF-1α were detected in the majority of chemo-resistant CC samples, and SOX2 expression was found to positively correlate with the expressions of USP7 and HIF-1α (Fig. 7A-B). Using FISH and immunofluorescence assay, we found that lnc-RP11-536 K7.3 and SOX2 was downregulated in chemosensitive tissues (Fig. 7C). Moreover, we discovered that the expression of SOX2, USP7, HIF-1α and the angiogenic marker CD31 were changed in accordance with lnc-RP11-536 K7.3 expression level both in chemo-resistant and chemo-sensitive human CC tissue sample (Fig. S6A-D). PET/CT revealed that SUVmax value was increased in chemo-resistant CC samples compared to that in chemo-sensitive CC samples (Fig. 7D-E). Besides, SUVmax value was found to positively correlate with lnc-RP11-536 K7.3 expression, and high SUVmax value predicted a poor prognosis (Fig. 7F). Besides that, low expression of CD31 was observed in chemosensitive CC tissues (Fig. 7G). The schematic model of how lnc-RP11-536 K7.3 participates in oxaliplatin resistance in patient-derived CC organoids and cells was illustrated (Fig. 7H).

## Discussion

Advanced or metastatic CC patients who develop resistance to oxaliplatin have a poor prognosis and a limited number of therapeutic options [17, 18]. It is therefore meaningful to understand the biological mechanism of resistance to oxaliplatin and identify novel therapeutic targets to enhance chemosensitivity. Moreover, identification of effective biomarkers contributes to the selection of oxaliplatin-responsive patients and realizes clinical benefits. In the current study, patient-derived organoids were exploited to profile molecular network and mimic therapeutic responses in oxaliplatin-resistant or -sensitive organoids of CC patients. Organoids retain the genetic and phenotypic stability of their original tissues to a great extent and have a superior predictive ability of patient-specific responses to chemotherapy, highlighting a great promise for personalized therapy [19, 20]. Different from previous studies that employed high-throughput RNA sequencing [21, 22] (), the present study adopted mass spectrometry-based proteomics to explore the molecular mechanism of oxaliplatin resistance. Ubiquitin-mediated proteolysis, carbon metabolism, and VEGF signaling pathway were identified as novel characteristics of oxaliplatin-resistant organoids of CC patients. This may motivate us to study the mechanism of oxaliplatin resistance from a new perspective.

It is noteworthy that lncRNAs are emerging as novel diagnostic, prognostic, and therapeutic targets in CC research compared to protein coding genes [23, 24]. In the present research, we identified a novel lncRNA, lnc-RP11-536 K7.3, to be highly expressed in oxaliplatin-resistant organoids of human CC tissues. To date, no research has concentrated on the functions and regulatory mechanisms of lnc-RP11-536 K7.3 Our data suggested that high expression of lnc-RP11-536 K7.3 in human CC tissues was significantly associated with a poor oxaliplatin response and an inferior prognosis. Therefore, it might be necessary to determine the expression of lnc-RP11-536 K7.3 in CC tissues to identify patients who might benefit more from oxaliplatin treatment before making a clinical decision. Our results revealed that downregulation of lnc-RP11-536 K7.3 could impede CC progression and resensitize CC to oxaliplatin treatment both in vitro and in vivo. Hence, we demonstrated that lnc-RP11-536 K7.3 exerted its functions at least partially through modulating glycolysis and angiogenesis. Emerging evidence suggested that chemo-resistant cancer cells exhibited a high level of glycolysis, and targeting glycolysis could be a promising strategy to reverse drug resistance [25–27]. However, the potential mechanisms triggering glycolysis modulation have largely remained elusive. Several molecules have been found to participate in this process [26, 28, 29]. For instance, knockdown of PTBP1 could attenuate the resistance of CC cells to oxaliplatin through regulation of glycolysis [30]. In the present research, we verified the role of lnc-RP11-536 K7.3 in modulating chemosensitivity via glycolysis and vascular remodeling, suggesting that targeting lnc-RP11-536 K7.3 is a promising therapeutic strategy for oxaliplatin-resistant organoids of CC patients in clinical setting.

Additionally, lncRNAs have been proposed to regulate local gene expression in *cis* or leave the site of transcription and perform transcriptional regulation in *trans*. lncRNA interacting with transcription factors to regulate tumor-related gene expression has been found as a major mechanism in *trans* [31, 32]. For example, lncRNA HNF1A-AS1 was demonstrated to bind to transcription factor PBX3 to upregulate OTX1 [33]. To elucidate the molecular mechanism of oxaliplatin resistance mediated by lnc-RP11-536 K7.3, RNA pull-down assay in combination with RIP assay was conducted, and SOX2 was identified as an interacting protein. Besides, lnc-RP11-536 K7.3 recruited SOX2 to transcriptionally activate USP7. Intriguingly, lnc-RP11-536 K7.3 not only bound to SOX2 and regulated detection of USP7 promoter, but also affected the expression of SOX2 itself. Therefore, the regulatory effect of lnc-RP11-536 K7.3 on SOX2 transcriptional activity might be a synergetic result of expression modulation, as well as substrate recognition. How lnc-RP11-536 K7.3 can regulate SOX2 expression remains unknown. Studies revealed a positive feedback loop between two transcription factors, SOX2 and SOX6, leading to mutually stimulation of expression [34]. Whether interaction between lnc-RP11-536 K7.3 and SOX2 stimulates SOX6 transcription and forms a positive feedback loop to regulate SOX2 expression should be further investigated.

Furthermore, we found that lnc-RP11-536 K7.3 could stabilize HIF-1α by upregulating USP7 expression. USP7, as a deubiquitinating enzyme, could regulate the stability of tumor-associated substrates [35–37]. It was reported that USP7 could activate Wnt signaling pathway and promote tumorigenesis by mediating β-catenin deubiquitination in adenomatous polyposis coli (APC) mutations from colon tumors [38]. In the present research, we identified HIF-1α as a putative substrate of USP7, and we demonstrated that knockdown of USP7 resulted in increased HIF-1α ubiquitination and destabilized HIF-1α protein. HIF-1α upregulation has been observed in a variety of human malignant diseases (e.g., CC) [9, 34, 39]. HIF-1α is involved in carcinogenesis, tumor angiogenesis, and cancer progression [40]. Additionally, HIF-1α plays a critical role in drug resistance, and targeting HIF-1α has been reported to significantly attenuate hypoxia-induced oxaliplatin resistance in CC [9].

## Conclusion

In summary, a novel lncRNA, lnc-RP11-536 K7.3, was identified to upregulate USP7 by recruiting SOX2. Upregulated USP7 deubiquitinates and stabilizes HIF-1α to maintain oxaliplatin resistance and to promote CC progression. Our findings demonstrate that lnc-RP11-536 K7.3 plays a crucial role in oxaliplatin resistance, and highlight its significance as a prognostic factor, a predictive indicator for oxaliplatin treatment, and a promising therapeutic target in CC.

## Supplementary Information

**Additional file 1: Table S1.** Association between lnc-RP11-536 K7.3 expression and clinicpathological factors in colon cancer TMA (*n* = 276).

**Additional file 2.** Supplementary Methods.

**Additional file 3: Figure S1.** Chemo-resistant and -sensitive organoids derived from CC patients. (A) Hematoxylin-eosin (HE) staining of oxaliplatin-resistant and -sensitive organoids of CC tissues (CR: Chemo-resistant; CS: Chemo-sensitive). (B) Images of oxaliplatin-resistant and -sensitive organoids derived from CC patients with or without 1 uM oxaliplatin treatment for 21 days. (C) Cell viability assay of organoids treated with 1 uM oxaliplatin in different time intervals. (D) HE staining of oxaliplatin-resistant and -sensitive organoids of CC patients. (E) Immunohistochemistry of CK20 and β-catenin in oxaliplatin-resistant and -sensitive organoids of CC patients.

**Additional file 4: Figure S2.** Immunohistochemistry staining of human colon cancer markers and the survival analysis of lnc-RP11-536 K7.3 in different cancer patients. (A) Immunohistochemistry staining of CK20 and β-catenin in oxaliplatin-resistant and -sensitive human colon cancer tissues. (B) Survival analysis of lnc-RP11-536 K7.3 in different cancer patients in TCGA database. (C) The expression of lnc-RP11-536 K7.3 in cancer tissues and the adjacent sites in TCGA database.

**Additional file 5: Figure S3.** Overexpressing lnc-RP11-536 K7.3 in the depleted cells/organoids restores glycolysis and angiogenesis. (A-D) Determination of glucose uptake (A), ATP (B), NADPH (C), and lactate production (D) in CC organoids and cells as indicated. Data were presented as mean ± SD of triplicate measurements repeated three times with similar results. Statistical significance was assessed via the Student’s t-test (^**^P < 0.01). (E-F) Measurement of ECAR (E) and OCR (F) in CC organoids and cells as indicated. (G) Statistical analysis of tube formation in different groups (^**^P < 0.01).

**Additional file 6: Figure S4.** Effect of lnc-RP11-536 K7.3 knockout, SOX2 overexpression on oxaliplatin-sensitivity, glycolysis and angiogenesis in chemo-resistant colon cancer organoids and cells. (A) Cell viability assay of chemo-resistant colon cancer organoids treated with 1uM oxaliplatin in different time intervals. (B) Values of IC50 of oxaliplatin. Colon cancer cells were treated with different. Concentration of oxaliplatin for 48 h. (C) Relative colony formation efficiency of chemo-resistant colon cancer organoids and cells treated with or without 2uM oxaliplatin for 7 days. (D) Determination of glucose uptake, ATP, NADPH and lactate production as described in Methods. Data are means ± SD of triplicate measurements repeated 3 times with similar results. Statistical significance was assessed with two-tailed Student’s t test. (E-F) ECAR (E) and OCR (F) were determined as described in Methods.

**Additional file 7: Figure S5.** Immunofluorescence assay of nude mice transplanted tumor tissues. (A) Immunofluorescence assay indicated the association between SOX2 and USP7 in nude mice transplanted tumor tissues. (B) Immunofluorescence assay indicated the association between SOX2 and CD31 in nude mice transplanted tumor tissues.

**Additional file 8: Figure S6.** Immunofluorescence assay of human colon tissues. (A) Immunofluorescence assay indicated the association between SOX2 and USP7 in lnc-RP11-536 K7.3 high expressed chemoresistant colon cancer tissues and lnc-RP11-536 K7.3 low expressed chemosensitive colon cancer samples. (B) Immunofluorescence assay indicated the association between SOX2 and HIF-1α in lnc-RP11-536 K7.3 high expressed chemoresistant colon cancer tissues and lnc-RP11-536 K7.3 low expressed chemosensitive colon cancer samples. (C) Immunofluorescence assay indicated the association between SOX2 and CD31 in lnc-RP11-536 K7.3 high expressed chemoresistant colon cancer tissues and lnc-RP11-536 K7.3 low expressed chemosensitive colon cancer samples. (D) Immunofluorescence assay indicated the association between USP7 and CD31 in lnc-RP11-536 K7.3 high expressed chemoresistant colon cancer tissues and lnc-RP11-536 K7.3 low expressed chemosensitive colon cancer samples.

## Data Availability

For all data requests, please contact the corresponding author.

## Abbreviations

CC: Colon cancer
LncRNAs: Long non-coding RNAs
FUSCC: Fudan University Shanghai Cancer Center
OS: Overall survival
PFS: Progression-free survival
ATCC: American Type Culture Collection
IHC: Immunohistochemistry
IRS: Immunoreactive score
GSEA: Gene Set Enrichment Analysis
qRT-PCR: Quantitative reverse transcription polymerase chain reaction
SDS-PAGE: Sodium dodecyl sulfate polyacrylamide gel electrophoresis
BSA: Bovine serum albumin
OCR: Oxygen consumption rate
ECAR: Extracellular acidification rate
PBS: Phosphate-buffered saline
CCK-8: Cell viability was examined with Cell Counting Kit-8
OD: Optical density
EGFP: Enhanced green fluorescent protein
HE: Hematoxylin-eosin
