# Supplementary material for: Lnc-RP11-536 K7.3/SOX2/HIF-1α signaling axis regulates oxaliplatin resistance in patient-derived colorectal cancer organoids
Source: J Exp Clin Cancer Res. 2021 Nov 5;40:348. doi: 10.1186/s13046-021-02143-x (PMC8570024; doi:10.1186/s13046-021-02143-x)
Supplement: Supplementary file 1 — Additional file 1: Table S1. Association between lnc-RP11-536 K7.3 expression and clinicpathological factors in colon cancer TMA (n = 276). [file 13046_2021_2143_MOESM1_ESM.docx]

**Table S1. Association between lnc-RP11-536K7.3 expression and clinicopathological factors in colorectal cancer TMA (n = 276)**

| **Variable** | **n** | **lnc-RP11-536K7.3 Expression** | | **χ^2^ Value** | **P value** |
| --- | --- | --- | --- | --- | --- |
|  |  | **Low** | **High** |  |  |
| **Gender** |  |  |  | 0.514 | 0.296 |
| Male | 166 | 51(30.72) | 115(69.28) |  |  |
| Female | 110 | 38(34.55) | 72(65.45) |  |  |
| **Age** |  |  |  | 0.002 | 0.962 |
| ≤60 | 170 | 35(32.4) | 115(67.6) |  |  |
| >60 | 106 | 34(32.1) | 72(67.9) |  |  |
| **Primary site** |  |  |  | 0.771 | 0.380 |
| Colon | 129 | 45(34.9) | 84(65.1) |  |  |
| Rectum | 147 | 44(29.9) | 103(70.1) |  |  |
| **Histological type** |  |  |  | 1.089 | 0.297 |
| Adenocarcinoma | 261 | 86(33.0) | 175(67.0) |  |  |
| Mucinous/SRCC* | 15 | 3(20.0) | 12(80.0) |  |  |
| **T category** |  |  |  | 24.605 | <0.001 |
| T1/T2 | 43 | 26(60.5) | 17(39.5) |  |  |
| T3 | 54 | 22 (40.7) | 32(59.3) |  |  |
| T4 | 179 | 41(22.9) | 138(77.1) |  |  |
| **N stage** |  |  |  | 58.077 | <0.001 |
| N0 | 120 | 68(56.7) | 52(43.3) |  |  |
| N1 | 83 | 10(12.2) | 72(87.8) |  |  |
| N2 | 73 | 11(14.9) | 63(85.1) |  |  |
| **M stage** |  |  |  | 5.687 | *0.017* |
| M0 | 234 | 82(35.0) | 152(65.0) |  |  |
| M1 | 15 | 7(16.7) | 35(83.3) |  |  |
| [**Pathological grading**](http://dict.cn/pathological%20grading) |  |  |  | 2.143 | 0.543 |
| High | 59 | 23(39.0) | 36(61.0) |  |  |
| Moderate | 185 | 36(30.3) | 129(69.7) |  |  |
| Poor/ [undifferentiation](http://dict.cn/undifferentiation) | 16 | 5(8.3) | 11(5.1) |  |  |
| Unknown | 16 | 4(25.0) | 12(75.0) |  |  |
| **Venous invasion** |  |  |  | 16.198 | <0.001 |
| Negative | 188 | 75(39.89) | 113(60.11) |  |  |
| Positive | 88 | 14(15.9) | 74(84.1) |  |  |
| **Perineural invasion** |  |  |  | 8.803 | 0.003 |
| Negative | 231 | 83(35.9) | 148(64.1) |  |  |
| Positive | 45 | 6(13.3) | 39(86.7) |  |  |
| **CEA** **(μl/ml)** |  |  |  | 7.124 | *0.028* |
| ≤5 | 170 | 61(35.9) | 109(64.1) |  |  |
| >5 | 95 | 22(23.2) | 73(76.8) |  |  |
| Unknown | 11 | 6(54.5) | 5(45.4) |  |  |

*SRCC: signet-ring cell carcinoma
