# Supplementary material for: Lnc-RP11-536 K7.3/SOX2/HIF-1α signaling axis regulates oxaliplatin resistance in patient-derived colorectal cancer organoids
Source: J Exp Clin Cancer Res. 2021 Nov 5;40:348. doi: 10.1186/s13046-021-02143-x (PMC8570024; doi:10.1186/s13046-021-02143-x)
Supplement: Supplementary file 2 — Additional file 2. Supplementary Methods. [file 13046_2021_2143_MOESM2_ESM.docx]

**Supplementary Methods**

**Cell culture**

RKO-CC cells, purchased from American Type Culture Collection (ATCC, Manassas, VA, USA), were cultured in DMEM containing 10% fetal bovine serum (FBS) (Invitrogen Life Technologies) at 37 °C in presence of 5% CO_2_.

**Generation of lnc–RP11-536K7.3-knockout (KO) organoids and cell lines**

CRISPR/Cas9 system was utilized to generate lnc–RP11-536K7.3-KO organoids and cell lines, in form of KO1-RP11-536K7.3 and KO2-RP11-536K7.3. Disruption of RP11-536K7.3 expression was confirmed by high-throughput RNA sequencing and quantitative reverse transcription polymerase chain reaction (qRT-PCR). Organoids and cells were then infected with the lentivirus. After 72 h, puromycin (2 μg/ml) was added for 3 days.

**Oxygen consumption rate (OCR) and extracellular acidification rate (ECAR)**

Cellular mitochondrial function was measured using the Seahorse XF Cell Mito Stress Test Kit and the Bioscience XF96 Extracellular Flux Analyzer, according to the manufacturers' instructions. Glycolytic capacity was determined using the Glycolysis Stress Test Kit in accordance with the manufacturer's instructions. Briefly, 4*10^4^ cells were seeded onto 96-well plates and incubated overnight. After washing the cells with Seahorse buffer, 175 mL of Seahorse buffer plus 25 mL each of 1 mmol/L oligomycin, 1 mmol/L FCCP, and 1 mmol/L rotenone were automatically injected to measure the OCR. Then, 25 mL each of 10 mmol/L glucose, 1 mmol/L oligomycin, and 100 mmol/L 2-deoxy-glucose were added to measure the ECAR. The values of OCR and ECAR were calculated after normalization to the cell number and were plotted as the mean (standard deviation (SD)).

**Glycolysis**

The glycolysis was examined using the Lactate Colorimetric Assay Kit (BioVision Co., Ltd., Beijing, China), Glucose Uptake Colorimetric Assay Kit (BioVision Co., Ltd.), Amplite Colorimetric NADPH Assay Kit (AAT Bioquest Inc., Sunnyvale, CA, USA), and ATP Assay Kit (Sigma-Aldrich, St. Louis, MO, USA) following the manufacturers' protocols.

**Mass spectrometry**

Proteins were reduced with 10 mM DTT at 56 ℃, followed by alkylation with 55 mM iodoacetamide at room temperature in the dark. Trypsin digestion was conducted overnight at 37 ℃ via gentle shaking, followed by peptide extraction using 1% trifluoroacetic acid in 50% acetonitrile. Samples were vacuum-dried and reconstituted in 0.1% formic acid for subsequent mass spectrometry. Treated samples were examined via nanoLC-MS/MS (nanoACQUITY UPLC and SYNAPT G2 HD mass spectrometer; Waters Corp., Milford, MA, USA). Then, mass spectrometry data were collected and processed with PLGS 2.4 software (Waters Corp.), and the resultant peak list was searched via the NCBI database with the MASCOT search engine.

**Zebrafish embryo**

Zebrafishes were treated with or without oxaliplatin for 24 h. To evaluate blood vessel formation, enhanced green fluorescent protein (EGFP) transgenic embryos were injected with 4 ng RP11-536K7.3 morpholino plasmid. At 24 hours post-fertilization (hpf), embryos were dechlorinated and anesthetized with 0.016% MS-222 (Sigma-Aldrich). Each zebrafish embryo was mounted with 3% methylcellulose on a depression slide for fluorescence microscopy.

The effects of RP11-536K7.3 on VEGFA expression were measured via qRT-PCR. Total RNA was extracted from 15 to 20 embryos per group with TRIzol reagent (Roche, Basel, Switzerland) and reversely transcribed using PrimeScript RT.

**RNA pull-down assay**

LncRNA-RP11-536K7.3 and its antisense RNA were transcribed from pLV-RP11-536K7.3 vector and biotin-labeled with the Biotin RNA Labeling Mix (Roche) and T7/SP6 RNA polymerase, treated with RNase-free DNase I (Roche), and purified with an RNeasy Mini Kit (Qiagen). One milligram of protein stably transfected with pLV-RP11-536K7.3 extracts was then mixed with 50 pmol of biotinylated RNA, incubated with streptavidin agarose beads (Invitrogen), and washed. The retrieved proteins were resolved by SDS-PAGE, and the specific bands were excised and analyzed by mass spectrometry and Western blotting.

**RNA immunoprecipitation (RIP) assay**

RIP assay was undertaken by using a Magna RIP RNA-Binding Protein Immunoprecipitation Kit (Millipore, Bedford, MA, USA) according to the manufacturer’s instructions. Antibody for RIP assay of SOX2 (Abcam, Cambridge, UK) was diluted (1:1000). Co-precipitated RNAs were detected by qRT-PCR.

**Immunohistochemistry (IHC)**

For IHC experiments, paraffin-embedded tissue was sliced into sections (thickness, 3-µm), and incubated at 4 °C overnight with primary antibodies and subsequently with peroxidase-labeled anti-rabbit (or anti-mouse) secondary antibodies for 1 h at 37 °C. Immunoreactive score (IRS) was determined as multiplicity of staining intensity and percentage of positively stained cells. Protein expression was marked as negative ($\leq$1+) or positive (>2+ to $\leq$3+).

**High-throughput RNA sequencing**

1 mg total RNA was isolated from CC organoids and treated with VAHTS mRNA Capture Beads (Vazyme Biotech Co., Ltd., Nanjing, China) to enrich polyAþ RNA before construction of RNA libraries. RNA library preparation was performed using the VAHTS mRNA-seq v2 Library Prep Kit Illumina (Illumina Inc., San Diego, CA, USA). Paired-end sequencing was carried out with Illumina HiSeq 3000 (Illumina Inc.). For computational analysis of RNA sequencing data, reads were aligned using the spliced read aligner HISAT2, which was supplied with the Ensemble Human Genome Assembly (Genome Reference Consortium GRCh38) as the reference genome. Gene expression level for each transcript was estimated as number of reads per kilobase of exon model per million mapped reads (RPKM). Gene Set Enrichment Analysis (GSEA) was used for functional annotation. Genes were considered significantly differentially expressed in cases where expression differed between two samples with fold-change > 2 and p-value < 0.05, as calculated with Cufflinks.

**Immunofluorescence assay**

Cells, organoids or frozen sections of CC tissues were fixed with 4% paraformaldehyde for 15 min and permeabilized with 0.3% Triton X-100 for 15 min, followed by blocking with 5% goat serum (Invitrogen Life Technologies, Carlsbad, CA, USA) for 1 h at room temperature. Next, sections were incubated with the appropriate primary antibodies overnight at 4°C, followed by the corresponding secondary antibodies (either FITC- or Texas red-conjugated donkey F(ab)2 fragments against mouse IgG or rabbit IgG (Jackson ImmunoResearch Laboratories Inc., West Grove, PA, USA)), and stained with 4′,6-diamidino-2-phenylindole (DAPI) (Invitrogen Life Technologies). Stained sections were imaged under a Leica SP5 confocal fluorescence microscope (Leica, Wetzlar, Germany).

**Plasmid construction and viral infection**

For generating stable cell lines, lentiviruses harboring specific shRNAs against USP7 (shUSP7) were transduced into CC cells. Lentiviruses were produced by co-transfection of HEK293T cells with recombinant lentivirus vectors and pPACK Packaging Plasmid Mix (System Biosciences, Palo Alto, CA, USA) using Megatran reagent (OriGene, Rockville, MD, USA). Lentiviral shRNA vectors were constructed by cloning short hairpin RNA fragments into pSIH-H1-Puro (System Biosciences) and lentiviral vectors for gene overexpression were obtained by inserting amplified gene fragments into pCDH (System Biosciences).

**Western blotting**

Protein extracts from organoids or cell lines were isolated via 8-12% sodium dodecyl sulfate polyacrylamide gel electrophoresis (SDS-PAGE) and electrotransferred onto 0.2-µm nitrocellulose membranes. After blocking for 1 h in bovine serum albumin (BSA), membranes were incubated with the appropriate primary antibodies at 4 °C overnight, followed by secondary antibodies. Antibodies of USP7(ab264422), HIF-1α(ab51608), P27(ab32034), and β-actin(ab8226) were gained form Abcam company. Horseradish peroxidase (HRP)-conjugated secondary antibodies were obtained from Abcam too. The primary antibodies were diluted as 1:1000 in our study and secondary antibodies were diluted as 1:2000 for use.

**Tube formation**

An *in vitro* tube formation assay was performed in Matrigel (BD Biosciences) according to the manufacturer's protocol. Briefly, 300 μL Matrigel was coated onto a 24-well plate (BD Biosciences) and layered with 200 μL medium containing 2*10^4^ cells. After 6 h of incubation, tube formation was observed under an Olympus microscope (Olympus, Tokyo, Japan).

**qRT-PCR**

For qRT-PCR, total RNA was extracted from cells or organoids with TRIzol reagent (Invitrogen) and reversely transcribed using the miScript Reverse Transcription Kit (Qiagen, Hilden, Germany). Quantification was performed with the QuantiTect Probe RT-PCR Kit (Qiagen, Germany). The 2^-ΔΔCT^ method was applied to calculate relative changes in gene expression. The sequences of the USP7 primer was as follows: forward: 5’-GGAAGCGGGAGATACAGATGA-3’; reverse: 5’-AAGGACCGACTCACTCAGTCT-3’.

**Colony formation assay**

Cells (1500 cells/dish) were seeded into 3.5-cm dishes and cultured in a humidified environment under 5% CO_2_ at 37 °C for 24 h. Cells were treated with or without oxaliplatin at 37 °C for 7 days. Subsequently, the cells were thrice washed with phosphate-buffered saline (PBS), followed by staining with 1 ml crystal violet solution for 10 min at room temperature. After washing with PBS, cell colonies were counted.

Organoids were harvested and dissociated into single cells. Subsequently, cells were washed with PBS, resuspended in Basement Membrane Extract (2 × 10^6^ cells/ml), and seeded into standard 96-well culture plates in triplicate. The cells were cultured in complete human organoid media. Complete media were replaced every three days and finally the number of colonies formed in each well was counted under a microscope on the seventh day.

**Cell and organoid viability assay**

Cell and organoid viability were examined with Cell Counting Kit-8 (CCK-8) (Dojindo Laboratories, Kyoto, Japan). RKO cells or cells derived from CC organoids were seeded into 96-well plates at a cell density of 5*10^3^ per well (organoids were planted in Matrigel). On the next day, cells were treated with various concentrations of oxaliplatin. After 48 h, the cells or organoids were incubated with 10 μl CCK-8 diluted in a normal culture medium at 37 ◦C for 2 h, and optical density (OD) was measured at wavelength of 450 nm. All experiments were conducted in triplicate.

**Luciferase reporter assay**

Cells were transfected with the CRE-luciferase (pGL3 vector), and luciferase activity was measured via the GloMax-Multi Microplate Reader (Promega, Madison, WI, USA). Renilla luciferase was used to normalize luciferase enzyme activity.

**Establishment of a mouse xenograft model**

Experimental procedures and animal care were undertaken following the Guidelines for Animal Experiments, and approved by the Ethics Committee of FUSCC. A mouse xenograft model was established based on subcutaneous or intraperitoneal injection of cells (~5*10^6^) into 4-week-old male BALB/c nude mice. The tumor volume was calculated using the following formula: V (volume) = L (length) $\times$ W (width)^2^ $\times0.52.$ Once an average tumor volume of 100 mm^3^ could be achieved, mice were intraperitoneally administered with oxaliplatin every 3 days. Tumor volume was measured every 3 days. Mice were ultimately sacrificed and all tumors were dissected and weighed. Tumor cells of nude mice were quantified using a luminescence imaging system.

**Immunoprecipitation (IP) assay**

IP assay was undertaken by using the Pierce Crosslink Immunoprecipitation Kit (Thermo Fisher Scientific, Waltham, MA, USA) in accordance with the manufacturer's protocol. Briefly, 10 μg antibody was covalently crosslinked with agarose A/G beads using DSS and incubated with 1 mg total cell lysates overnight at 4 °C. Antigen was eluted and subjected to SDS-PAGE. Cell lysates were incubated with anti-FLAG M2 agarose beads or anti-Xpress agarose beads for 2-4 h. For IP of endogenous HIF-1α, cell lysates were primarily pre-cleared with A/G plus Sepharose. Then, 10 μg of HIF-1α antibody was incubated with pre-cleared cell lysates overnight. Finally, the antibody complex was incubated with A/G plus Sepharose for 1 h. Immunoprecipitants were washed four times with NETN buffer (20 mM Tris, pH 8.0, 100 mM NaCl, 1 mM EDTA, and 0.5% NP-40) before being resolved by SDS–PAGE.
