# Supplementary figures and images for: Lnc-RP11-536 K7.3/SOX2/HIF-1α signaling axis regulates oxaliplatin resistance in patient-derived colorectal cancer organoids
Source: J Exp Clin Cancer Res. 2021 Nov 5;40:348. doi: 10.1186/s13046-021-02143-x (PMC8570024; doi:10.1186/s13046-021-02143-x)

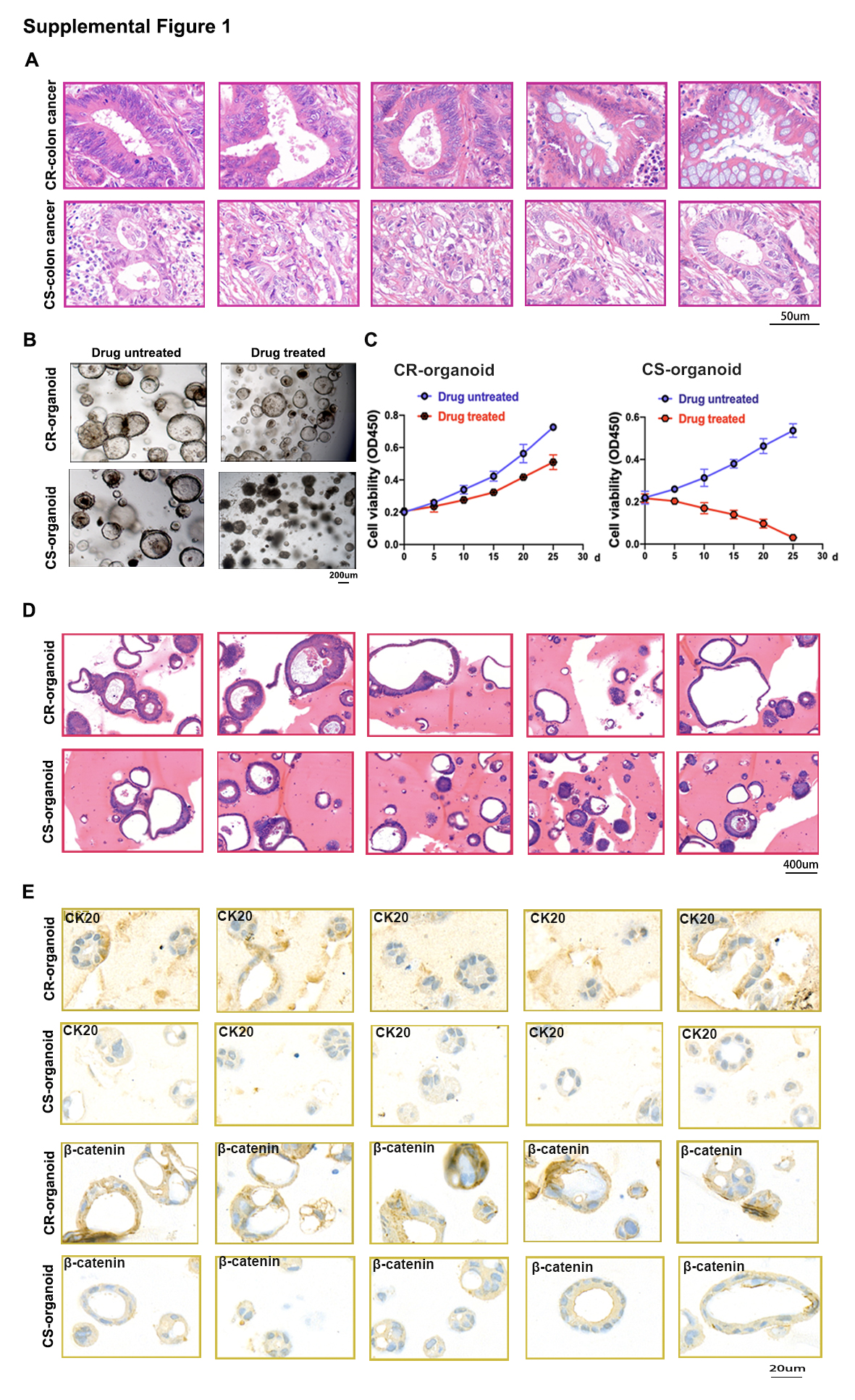

Supplement: Supplementary file 3 — Additional file 3: Figure S1. Chemo-resistant and -sensitive organoids derived from CC patients. (A) Hematoxylin-eosin (HE) staining of oxaliplatin-resistant and -sensitive organoids of CC tissues (CR: Chemo-resistant; CS: Chemo-sensitive). (B) Images of oxaliplatin-resistant and -sensitive organoids derived from CC patients with or without 1 uM oxaliplatin treatment for 21 days. (C) Cell viability assay of organoids treated with 1 uM oxaliplatin in different time intervals. (D) HE staining of oxaliplatin-resistant and -sensitive organoids of CC patients. (E) Immunohistochemistry of CK20 and β-catenin in oxaliplatin-resistant and -sensitive organoids of CC patients. [file 13046_2021_2143_MOESM3_ESM.jpg]

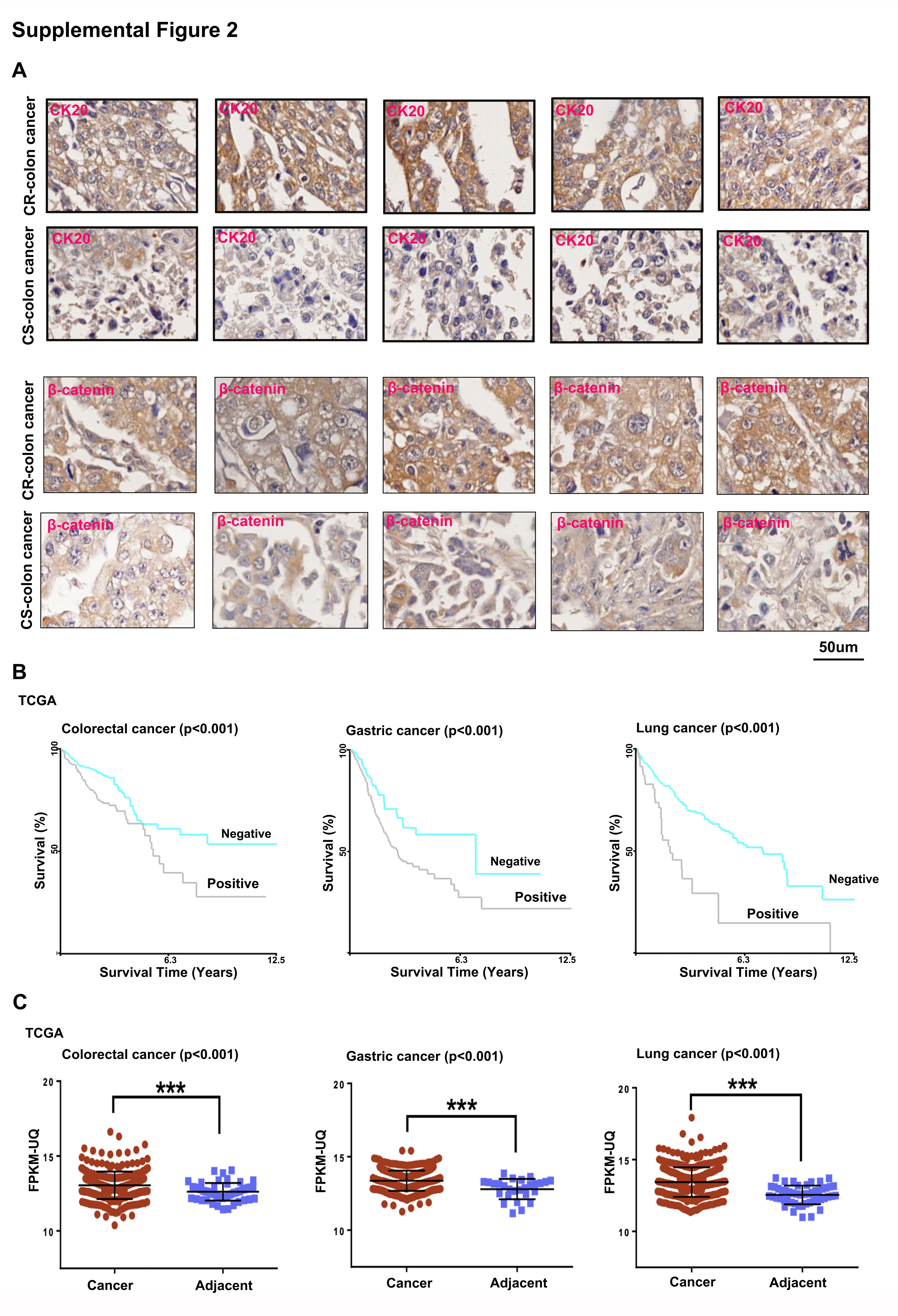

Supplement: Supplementary file 4 — Additional file 4: Figure S2. Immunohistochemistry staining of human colon cancer markers and the survival analysis of lnc-RP11-536 K7.3 in different cancer patients. (A) Immunohistochemistry staining of CK20 and β-catenin in oxaliplatin-resistant and -sensitive human colon cancer tissues. (B) Survival analysis of lnc-RP11-536 K7.3 in different cancer patients in TCGA database. (C) The expression of lnc-RP11-536 K7.3 in cancer tissues and the adjacent sites in TCGA database. [file 13046_2021_2143_MOESM4_ESM.jpg]

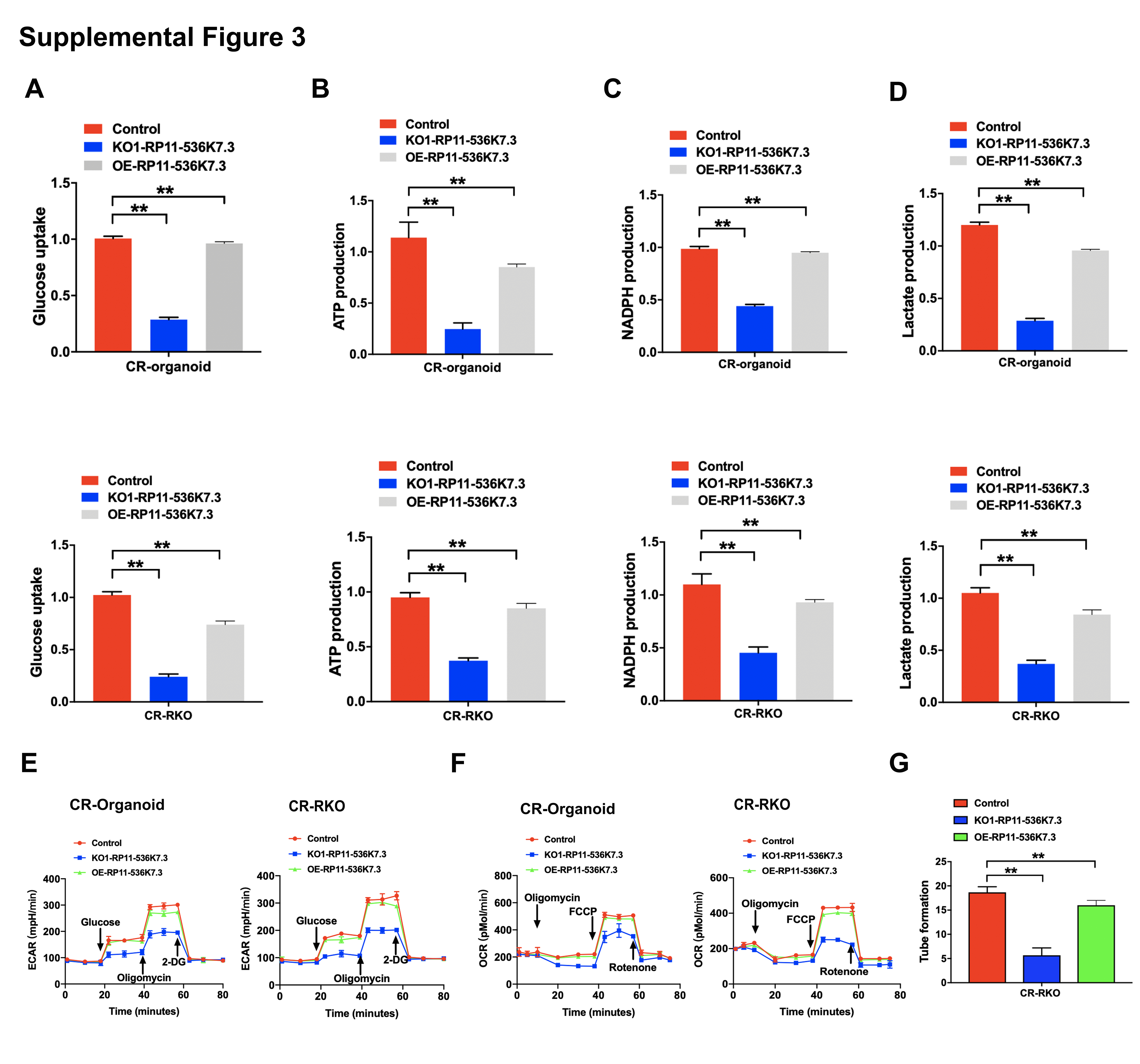

Supplement: Supplementary file 5 — Additional file 5: Figure S3. Overexpressing lnc-RP11-536 K7.3 in the depleted cells/organoids restores glycolysis and angiogenesis. (A-D) Determination of glucose uptake (A), ATP (B), NADPH (C), and lactate production (D) in CC organoids and cells as indicated. Data were presented as mean ± SD of triplicate measurements repeated three times with similar results. Statistical significance was assessed via the Student’s t-test (**P < 0.01). (E-F) Measurement of ECAR (E) and OCR (F) in CC organoids and cells as indicated. (G) Statistical analysis of tube formation in different groups (**P < 0.01). [file 13046_2021_2143_MOESM5_ESM.jpg]

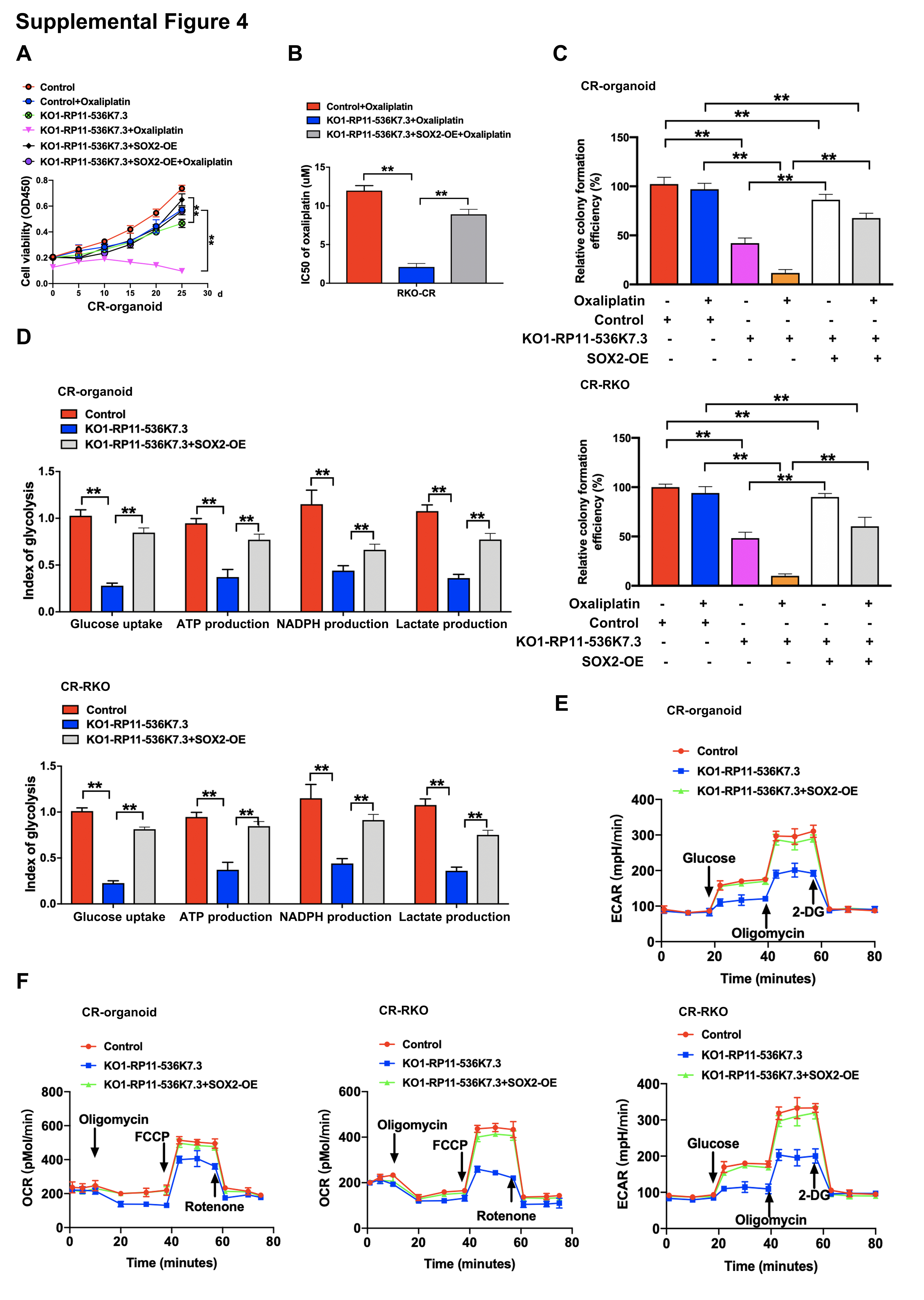

Supplement: Supplementary file 6 — Additional file 6: Figure S4. Effect of lnc-RP11-536 K7.3 knockout, SOX2 overexpression on oxaliplatin-sensitivity, glycolysis and angiogenesis in chemo-resistant colon cancer organoids and cells. (A) Cell viability assay of chemo-resistant colon cancer organoids treated with 1uM oxaliplatin in different time intervals. (B) Values of IC50 of oxaliplatin. Colon cancer cells were treated with different. Concentration of oxaliplatin for 48 h. (C) Relative colony formation efficiency of chemo-resistant colon cancer organoids and cells treated with or without 2uM oxaliplatin for 7 days. (D) Determination of glucose uptake, ATP, NADPH and lactate production as described in Methods. Data are means ± SD of triplicate measurements repeated 3 times with similar results. Statistical significance was assessed with two-tailed Student’s t test. (E-F) ECAR (E) and OCR (F) were determined as described in Methods. [file 13046_2021_2143_MOESM6_ESM.jpg]
